# Multiple aspects of energy poverty are associated with lower mental health-related quality of life: A modelling study in three peri-urban African communities

**DOI:** 10.1016/j.ssmmh.2022.100103

**Published:** 2022-12

**Authors:** Matthew Shupler, Miranda Baame, Emily Nix, Theresa Tawiah, Federico Lorenzetti, Jason Saah, Rachel Anderson de Cuevas, Edna Sang, Elisa Puzzolo, Judith Mangeni, Emmanuel Betang, Mieks Twumasi, Seeba Amenga-Etego, Reginald Quansah, Bertrand Mbatchou, Diana Menya, Kwaku Poku Asante, Daniel Pope

**Affiliations:** a Department of Public Health, Policy and Systems, University of Liverpool, Liverpool, United Kingdom; b Douala General Hospital, Douala, Cameroon; c Kintampo Health Research Centre, Research and Development Division, Ghana Health Service, Kintampo North Municipality, Ghana; d School of Public Health, Moi University, Eldoret, Kenya; e Global LPG Partnership (GLPGP), 654 Madison Avenue, New York, United States; f School of Public Health, University of Ghana, Ghana

**Keywords:** SF-36, Health-related quality of life, Energy poverty, Electricity, Clean cooking, LPG, Burns

## Abstract

**Objective:**

Over 900 million people in sub-Saharan Africa (SSA) live in energy poverty, relying on cooking polluting fuels (e.g. wood, charcoal). The association between energy poverty and mental/physical health-related quality of life (HRQoL) among women in SSA, who are primarily tasked with cooking, is unknown.

**Methods:**

Females (n ​= ​1,150) from peri-urban Cameroon, Kenya and Ghana were surveyed on their household energy use and mental/physical health status using the standardized Short-Form 36 (SF-36) questionnaire. Random effects linear regression linked household energy factors to SF-36 mental (MCS) and physical component summary (PCS) scores. A binary outcome of ‘likely depression’ was derived based on participants' MCS score. Random effects Poisson regression with robust error variance assessed the relationship between household energy factors and odds of likely depression.

**Results:**

The prevalence of likely depression varied by a factor of four among communities (36%-Mbalmayo, Cameroon; 20%-Eldoret, Kenya; 9%-Obuasi, Ghana). In the Poisson model (coefficient of determination (R^2^) ​= ​0.28), females sustaining 2 or more cooking-related burns during the previous year had 2.7 (95%CI:[1.8,4.1]) times the odds of likely depression as those not burned. Females cooking primarily with charcoal and wood had 1.6 times (95%CI:[0.9,2.7]) and 1.5 times (95%CI:[0.8,3.0]) the odds of likely depression, respectively, as those primarily using liquefied petroleum gas. Women without electricity access had 1.4 (95%CI:[1.1,1.9]) times the odds of likely depression as those with access. In the MCS model (R^2^ ​= ​0.23), longer time spent cooking was associated with a lower average MCS score in a monotonically increasing manner. In the PCS model (R^2^ ​= ​0.32), women injured during cooking fuel collection had significantly lower (−4.8 95%CI:[-8.1,-1.4]) PCS scores.

**Conclusion:**

The burden of energy poverty in peri-urban communities in SSA extends beyond physical conditions. Experiencing cooking-related burns, using polluting fuels for cooking or lighting and spending more time cooking are potential risk factors for lower mental HRQoL among women.

## Highlights

• Higher SF-36 scores among females/males cooking with LPG versus polluting fuels.
• Those cooking >10 ​h/week had poorer mental well-being than those cooking <5 ​h.
• 11 times odds of depression among females with 2+ burns compared to those not burned.
• Electricity access associated with lower odds of likely depression among females.
• Prevalence of likely depression varied by a factor of four between the three communities.

## Graphical abstract

**Figure:**
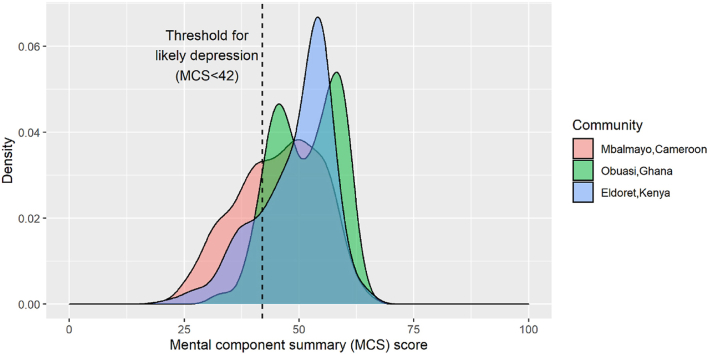

## 1 Introduction

Mental illness (e.g. anxiety and depression) accounts for approximately 15% of years lived with disability in low- and middle-income countries (LMICs) (Murray et al., 2020). The causes for the high burden of poor mental health are multifactorial, and are determined by both personal behaviors and environmental exposures (Helbich, 2018). Identifying amenable, environmental risk factors for mental illness at a population-level in LMICs is important for helping lower this burden (Collins et al., 2011). Research on environmental risk factors of poor mental health, such as air pollution exposure, has been mostly conducted in high-income countries (Power et al., 2015; Pun et al., 2017; Kioumourtzoglou et al., 2017; Bakolis et al., 2020; Braithwaite et al., 1260); few epidemiological studies have evaluated environmental risk factors of poorer mental health in LMICs. As environmental risk factors vary greatly between high and LMICs, there is a need for additional studies of mental health determinants in resource-poor settings (Lin et al., 2017; Winter et al., 2019).

### 1.1 Energy poverty as a determinant of poor mental health

Energy poverty, or fuel poverty, has been associated with poorer well-being in high income countries (Davillas et al., 2022). In LMICs, energy poverty, defined in this study as a lack of access to affordable, modern, clean fuels (e.g. liquefied petroleum gas (LPG), electricity) for cooking, heating or lighting is highly prevalent; approximately 3.8 billion people are dependent on polluting cooking fuels (e.g. wood, charcoal) (Health Effects Institute, 2020). Sub-Saharan Africa has the highest population-level reliance on polluting cooking fuels (approximately 85% of the population (Health Effects Institute, 2020)), equating to 900 million individuals lacking access to clean cooking fuels on the continent (Stoner et al., 2021). Given that approximately 30% of the population in sub-Saharan Africa (SSA) have been found to be depressed or anxious (Sweetland et al., 2019), and that a 130% increase in mental and substance use disorders is predicted in the region by 2050 (Charlson et al., 2014), investigating the relationship between indicators of energy poverty and mental health may uncover new strategies for addressing the rising burden of mental illness.

Individuals living in energy poverty are continuously exposed to high levels of household air pollution (HAP) from incomplete combustion of polluting fuels burned in inefficient stoves. HAP exposure leads to oxidative stress and systemic inflammation in the body (Ng et al., 2008; Vogelzangs et al., 2013), which can cause adverse physical health outcomes (Brook, 2004; Brook et al., 2010). It is possible that HAP exposure may adversely affect mental health via the same underlying mechanism (Buoli et al., 2018; Ehsanifar et al., 2022). Energy poverty may further negatively affect mental health via multiple lifestyle pathways, including productivity, education, food security, income and access to clean water, sanitation and medical care, among others (Winter et al., 2019; Bridge et al., 2016). Moreover, cooking with polluting fuels can lead to ‘time poverty’ arising from longer cooking times and need for frequent biomass fuel collection, potentially leading to chronic exhaustion and stress (Breitkreuz et al., 2010). Energy poverty can further lead to psychological stress from unstable cooking fuel prices and fluctuations in polluting fuel supply, or a consistent worry about the inability to cover energy costs (Dzator, 2013). Because women are traditionally the main cook in LMICs, energy/time poverty may disproportionately negatively impact their mental health (Haddad et al., 2021).

Studies from rapidly urbanising countries including China (Liu et al., 2020; Shao et al., 2021) and India (Banerjee et al., 2012) have found links between cooking with polluting fuels and depression or lower well-being. Yet, despite the multidimensional nature of energy poverty (Zhang et al., 2021), limited quantitative studies have simultaneously evaluated if multiple aspects of energy poverty, including reliance on polluting cooking fuels, suffering from related harms (injury while gathering fuels or burns during cooking), longer time spent cooking and lack of access to electricity for lighting, are associated with poorer mental well-being. We hypothesize that many of these factors will independently contribute to poorer mental health in SSA.

### 1.2 Using health-related quality of life instruments to assess mental health

As mental illness is often underdiagnosed in SSA due to generally lower mental health literacy among healthcare professionals and higher perceived stigma associated with mood and anxiety disorders (Sankoh et al., 2018), identifying determinants of poor mental health in SSA can be particularly challenging. Likert scale instruments that assess health-related quality of life (HRQoL), an individual's satisfaction with their daily functioning, life-satisfaction and ability to develop mutually beneficial relationships, have been successfully used to assess mental health across multiple populations (Ware and Gandek, 1998; Ware, 2000), including in SSA (Wyss et al., 1999; Patel et al., 2017; Kuo and Operario, 2011; Verduin et al., 2014; Lambert et al., 2021). Mental and physical HRQoL (or well-being), defined as a person's ‘perception of their position in life in the context of the culture and value systems in which they live, and in relation to their goals, expectations, standards and concerns’ (World Health Organization, 1997), has been found to be highly associated with depression (Moore et al., 2005) and severity of depression (IsHak et al., 2011). This study therefore aims to examine if several indicators of energy poverty are associated with poorer mental and physical HRQoL among female primary cooks in three peri-urban communities in SSA.

## 2 Methods

### 2.1 Study setting and population

This study is part of the *CLEAN-Air(Africa)* program, which includes research in three SSA countries: Kenya, Cameroon and Ghana. These three countries were specifically chosen to participate in *CLEAN-Air(Africa)* since the governments are seeking to increase LPG use to decrease the negative health and environmental impacts of reliance on polluting cooking fuels (Bruce et al., 2018). Peri-urban communities (Mbalmayo, Central Cameroon; Eldoret, Western Kenya; Obuasi, Ashanti Region, Ghana) were selected from each country to capture a mix of households primarily cooking with polluting fuels and LPG. Mbalmayo is an agricultural town in central Cameroon with approximately 70,000 residents that is a suburb (hour drive away) of Yaoundé, the country's capital. Obuasi is a gold-mining town in southern Ashanti, Ghana with a population of around 200,000 and is similarly an hour drive away from urban Kumasi (capital city of the Ashanti region). Eldoret is also an agricultural town located at an elevation of over 2000 ​m in Western Kenya with a population of nearly 500,000.

Phase 1 of CLEAN-Air(Africa) consisted of a population-based survey of 2,000 participants in each community (∼6,000 individuals total) in 2019 (Shupler et al., 2021). Stratified random sampling was used to select 400 primary cooks (200 primarily cooking with LPG and 200 exclusively cooking with polluting biomass fuels) in each community to participate in a second survey (Phase 2). Here, we report Phase 2 survey findings, which included additional questions on household energy use, cooking behaviours (including questions from the WHO harmonized household energy survey) (World Health Organization, 2019) and an HRQoL assessment (The Medical Outcomes Study Short Form-36 (SF-36), explained below in detail). A total of 1,240 primary cooks completed the Phase 2 survey (∼40 ​min to complete). Surveys were translated into the local language in each community (Cameroon: French; Kenya: Kiswahili; Ghana: no translation (English)) by study coordinators and administered by local field teams using smartphones/tablets. The data was encrypted using secure web technology (Mobenzi Researcher in Cameroon/Kenya; Research Electronic Data Capture (REDCap) in Ghana).

### 2.2 Outcome variables

The SF-36 questionnaire is the gold standard for assessing HRQoL. The SF-36 has been administered to various populations across different cultural settings in over 60 languages and more than 7,000 studies, including in SSA (Wyss et al., 1999; Patel et al., 2017; Kuo and Operario, 2011; Verduin et al., 2014; Lambert et al., 2021). Reliability studies of the SF-36 in SSA have demonstrated high internal consistency (Cronbach's alpha >0.80) (Wyss et al., 1999; Patel et al., 2017; Kuo and Operario, 2011; Verduin et al., 2014; Lambert et al., 2021). The SF-36 is separated into eight domains: general health perception, physical functioning, role-physical functioning, bodily pain, vitality, social functioning, role-emotional functioning and mental health. Descriptions of the questions included in eight domains of the SF-36 and how they are scored are provided in Supplementary Fig. 1 and Supplementary Tables 1 and 2, respectively (Ware et al., 1993).

The main outcomes in this study are the SF-36 physical component summary (PCS) and mental component summary (MCS) scores, which form two summary constructs (physical and mental health, respectively). These measures were chosen as they condense the eight SF-36 domains into two, thereby lowering the probability of an erroneous inference by reducing the number of statistical comparisons. The PCS score includes four domains: physical functioning, role-physical functioning, bodily pain, and general health. The MCS score consists of the other four domains: vitality, social functioning, role-emotional functioning and mental health domains. The PCS and MCS scores are calculated in five steps:

1) Using norm-based scoring algorithms, the individual SF-36 questions (full list of questions: https://www.rand.org/health-care/surveys_tools/mos/36-item-short-form/survey-instrument.html) are assigned a numeric value from 0 (lowest) to 100 (highest), representing the percentage of total possible score achieved (lower MCS and PCS scores indicate poorer HRQoL) (Supplementary Table 1). Questions have different scoring systems based on the number of multiple-choice options.
2) Scores from questions within the same domain are averaged together to generate the eight domain scores (e.g. social functioning domain score) (Supplementary Table 2).
3) The eight domain scores are standardized using a linear z-score transformation. Z-scores are derived by subtracting the mean domain score from the U.S. general population from each participant's score and dividing by the standard deviation of the U.S. population (Taft et al., 2001).
4) The eight domain z-scores are multiplied by corresponding factor ‘score coefficients’ supplied by the Medical Outcomes study and derived from an orthogonal model that assumes physical and mental health are uncorrelated (Taft et al., 2001). The score coefficients (also shown in Table 1 of Taft et al. (2001)) differ for the PCS and MCS score (Equation 1).

**Table 1:** Socioeconomic and cooking environment characteristics of female participants by community.

Characteristic	Overall (N ​= ​1,157)	Mbalmayo, Cameroon (N ​= ​404)	Obuasi, Ghana (N ​= ​348)	Eldoret, Kenya (N ​= ​405)
Age of household head (Mean (SD))	35 (12)	36 (13)	36 (10)	35 (12)
Fuel decision maker of household Yes	884 (76%)	225 (56%)	335 (96%)	320 (79%)
Head of household				
Yes	322 (26%)	103 (24%)	116 (33%)	103 (24%)
Marital status				
Married	652 (56%)	159 (39%)	211 (61%)	282 (70%)
Single	251 (22%)	97 (24%)	62 (18%)	92 (23%)
Cohabitating	152 (13%)	108 (27%)	44 (13%)	0
Widowed/divorced	95 (8%)	35 (9%)	29 (8%)	31 (7%)
Household size (# of members)				
1–2	118 (10%)	13 (3%)	67 (19%)	38 (9%)
3–4	369 (32%)	91 (22%)	120 (34%)	158 (39%)
5–6	382 (33%)	134 (33%)	110 (32%)	138 (34%)
7+	281 (24%)	161 (40%)	49 (14%)	71 (18%)
Financial security				
Have enough money	287 (23%)	40 (10%)	132 (38%)	96 (24%)
Not quite enough money	606 (49%)	198 (49%)	137 (39%)	237 (58%)
Definitely not enough money	330 (27%)	161 (41%)	77 (23%)	72 (18%)
Highest education level				
No formal education	58 (5%)	5 (1%)	39 (11%)	14 (3%)
Primary	296 (26%)	102 (25%)	72 (21%)	122 (30%)
Secondary/high school	604 (52%)	255 (63%)	223 (64%)	126 (31%)
University	192 (17%)	37 (9%)	12 (3%)	143 (35%)
Toilet in home				
Yes	384 (33%)	140 (35%)	90 (26%)	154 (38%)
Owns land				
Yes	426 (37%)	58 (15%)	108 (31%)	260 (64%)
Electricity connection				
Yes	837 (72%)	237 (59%)	323 (93%)	277 (68%)
Primary cooking fuel type				
LPG	527 (46%)	177 (45%)	166 (47%)	184 (45%)
Wood	396 (34%)	222 (55%)	31 (9%)	143 (35%)
Charcoal	227 (20%)	0	149 (43%)	78 (20%)
Hours cooking per week				
0.1–5	282 (24%)	44 (11%)	32 (9%)	204 (50%)
5.1–10	286 (24%)	106 (26%)	86 (25%)	91 (22%)
10.1–15	329 (30%)	141 (35%)	124 (36%)	63 (15%)
15.1–37	260 (22%)	108 (27%)	104 (30%)	47 (12%)
# of burns while cooking in last year				
1	27 (2%)	18 (5%)	9 (3%)	1 (0%)
2+	88 (8%)	74 (19%)	2 (0%)	12 (3%)
Obtain any cooking fuels for free				
Yes	178 (15%)	58 (15%)	26 (8%)	94 (24%)
Fuel collection frequency/month (n ​= ​178)				
1	66 (36%)	15 (26%)	0	51 (54%)
2–5	62 (34%)	31 (53%)	11 (42%)	20 (21%)
6+	50 (27%)	12 (21%)	15 (58%)	23 (25%)
Injury collecting fuels in last year (n ​= ​184)				
Yes	23 (13%)	9 (15%)	4 (15%)	10 (10%)
5) The domain specific z-scores are summed together separately for the PCS and MCS. Finally, the PCS and MCS sums are linearly transformed to norm-based PCS and MCS scores by multiplying by 10 and adding 50. This yields a mean of 50 and standard deviation of 10 (range from 0 to 100) for the U.S. general population (Ware et al., 1993). The U.S. factor score coefficients are used in this study to facilitate standardized comparisons between different study populations. These values (Transformed_MCS and Transformed_PCS in Equation 1, respectively) were the outcome measures used in this study.

MCS ​= ​ER_Z_SCORE∗0.43407 + SF_Z_SCORE∗0.26876 + VI_Z_SCORE∗0.23534 +

MH_Z_SCORE∗0.48581 + GH_Z_SCORE∗-0.01571 + PF_Z_SCORE∗-0.22999 +

PR_Z_SCORE∗-0.12329 + BP_Z_SCORE∗-0.09731

Transformed_MCS ​= ​50 + MCS∗10

PCS ​= ​ER_Z_SCORE∗-0.19206 + SF_Z_SCORE∗-0.00753 + VI_Z_SCORE∗0.02877 +

MH_Z_SCORE∗-0.22069 + GH_Z_SCORE∗0.24954 + PF_Z_SCORE∗0.42402 +

PR_Z_SCORE∗0.35119 + BP_Z_SCORE∗0.31754

Transformed_PCS ​= ​50 + PCS∗10

Equation 1. Calculation of MCS and PCS scores. ER ​= ​emotional role functioning; SF ​= ​social functioning; VI ​= ​vitality; MH ​= ​mental health; GH ​= ​general health; PF ​= ​physical functioning; PR ​= ​physical role functioning; BP ​= ​bodily pain.

Research conducted by the SF-36 instrument creators found that individuals’ scores do not typically fluctuate more than 6.5 points either direction, reflecting the 95% confidence level (2 standard deviations) for an expected change (Ware et al., 1996). Previous research has also found an average increase of at least 6.5 points in PCS score to occur after heart valve replacement and hip replacement surgeries, and be associated with a 33% decrease in the probability of job loss within the next year among working patients (see Tables 7.15 and 7.16 from (Ware et al., 1993)). This has led to a 6.5-point threshold being used as a metric for clinical and social importance in epidemiological studies (Bayliss et al., 2004).

The SF-36 MCS score has also been strongly correlated with severity of depression in general population studies (Beusterien et al., 1996). An MCS score of 42 has been established as a threshold for likely depression among older women; it was shown to reasonably well classify (sensitivity:71%; specificity:82%) a diagnosis (Silveira et al., 2005). Thus, in this study we additionally evaluated a binary outcome of ‘probable clinical depression’ among participants based on whether they scored at/above or below a 42 on the MCS scale. As a mental health domain score threshold of 52 has further been reported to be indicative of likely depression (Silveira et al., 2005), we conducted a sensitivity analysis to estimate the prevalence of likely depression using this criterion.

### 2.3 Explanatory variables

Explanatory variables included several indicators of energy poverty, including ‘primary’ cooking fuel type (‘What does this household use for cooking most of the time, including cooking food, making tea/coffee, boiling drinking water?’), authority over the type of cooking fuel used by the household (‘Who in your household makes the decision on what fuel is used or purchased for cooking?’), presence of an electricity connection (yes/no), method of obtaining the cooking fuel (purchase or gather for free), frequency of cooking fuel collection, suffering any injuries during fuel gathering and the number of times burned while cooking during the previous year. Energy poverty indicators were included together in a single regression model. A sensitivity analysis was conducted accounting for both primary and secondary cooking fuel type as an explanatory variable. In this analysis, participants using charcoal or wood as a primary fuel and LPG as a secondary cooking fuel were separated from those exclusively cooking with polluting fuels to determine if minimal use of LPG offered an improvement in MCS and PCS scores relative to no LPG use.

### 2.4 Covariates

Covariates were chosen for inclusion in the modelling based on a priori beliefs about their association with the exposure and outcome. Several sociodemographic variables (e.g. age, education level, level of self-reported level of financial security (enough money to support family, not quite enough, definitely not enough), marital status, primary water source for household tasks, land ownership, car ownership) were included. Due to 24% (n ​= ​290) of participants not wanting to disclose their household income, only the subjective measure of ‘financial security’ was used. Land ownership was considered as a proxy of food security as studies have shown that land ownership is a protective factor in the relationship between food security and poor mental health (Sweetland et al., 2019).

Health-related variables (e.g. smoking in the household, alcohol consumption) were also considered in the modelling. Participants were asked if they had been diagnosed with a medical condition, including tuberculosis, chronic bronchitis, heart disease and high blood pressure; a derived variable was generated based on whether a participant indicated a diagnosis of at least of these conditions or none. Where possible, continuous covariates were categorized to facilitate easier interpretation of model results; information on categorization of all predictor variables is presented in Supplementary Table 3.

### 2.5 Statistical analysis

Data management/analysis was conducted using R version 4.1.1 (R Core Team, 2017). Separate linear random effects regression models were used to evaluate the relationship between energy poverty and MCS and PCS scores, accounting for unmeasured similarities between participants within the same community. Linear fixed effects models were also built to compare to the coefficients from the linear random effects regression models.

The explanatory variables (energy poverty indicators) were first entered in the regression models one at a time and retained if they reduced the AIC. Certain sociodemographic variables known to confound the relationship between energy poverty and HRQoL (e.g. education, financial security, age) were retained in the models regardless of their impact on model fit. The remaining sociodemographic variables were added individually and remained in the model only if the AIC improved. Model optimization was performed using the coefficient of determination (R^2^) and AIC; cross validation was conducted to check for model overfitting.

Given that household income has previously been shown to modify the relationship between energy poverty and mental well-being in Ghana (Lin and Okyere, 2021), it was examined as an interaction term in the models. Interactions by community were also considered to test whether the association between energy poverty and well-being varied among the three study settings.

The prevalence of likely depression in each community was estimated and compared using the pre-established percent tile cut-off for the MCS score (42nd percent tile) and mental health domain score (52 percent tile) (Silveira et al., 2005). To assess the relationship between energy poverty and likely depression (based on an MCS score below the 42nd percent tile), we used Poisson regression with robust error variance via the “sandwich” package in R (Zeileis et al., 2020). Modified Poisson regression was used to avoid overestimating an association from logistic regression in cross-sectional studies with common outcomes (Barros and Hirakata, 2003; McNutt et al., 2003).

Due to a low number of male primary cooks (n ​= ​58), only female participants (n ​= ​1,150) were included in the modelling of MCS and PCS scores. Factors associated with self-perceived mental and physical HRQoL among males are presented descriptively. As approximately 10% (n ​= ​112) of female participants in Mbalmayo, Cameroon were missing measurement data on body mass index (BMI) (logistical issues during field work), BMI was not included in the main modelling. A sensitivity analysis was conducted that included BMI as a predictor (n ​= ​958).

### 2.6 Ethical approval

Ethical approval for this study was obtained from the University of Liverpool, United Kingdom and local ethics committees in each study country: Central Regional Ethics Committee for Human Health Research (Cameroon), Institutional Research and Ethics Committee for Moi Teaching and Referral Hospital and Moi University (Kenya) and Kintampo Health Research Centre Institutional Ethic Committee and Ghana Health Service Ethics Review Board (Ghana).

## 3 Results

The average age of female study participants was 35 years. In Mbalmayo, slightly over half (56%) the participants oversaw decision-making for the cooking fuels for the household, compared with nearly all participants (96%) in Obuasi (Table 1). Half (53%) of the participants received secondary school education. The proportion of participants reporting having ‘financial security’ (enough money to meet family needs) in Obuasi (38%) and Eldoret (24%) was four times and twice as high as in Mbalmayo (10%), respectively. Access to grid electricity was also lower in Mbalmayo (59%) than in Eldoret (70%) and Obuasi (94%).

Due to the stratified sampling, there was an approximately even distribution between participants primarily cooking with LPG (47%) and polluting fuels (wood, charcoal) (53%). The average cooking time during the week among households primarily cooking LPG was 9.96 ​h/week (SD:6.36), compared with 11.72 ​h/week (SD:6.47) among those primarily cooking with polluting fuels. A total of 115 (10%) female participants suffered at least one burn while cooking in the last year, with 88 (8%) having been burned more than once. In Mbalmayo, Cameroon, the prevalence of participants experiencing at least one cooking-related burn (23%) was eight times the proportion in Eldoret (3%) and Obuasi (3%).

### 3.1 SF-36 descriptive results

**Fig. 1:**
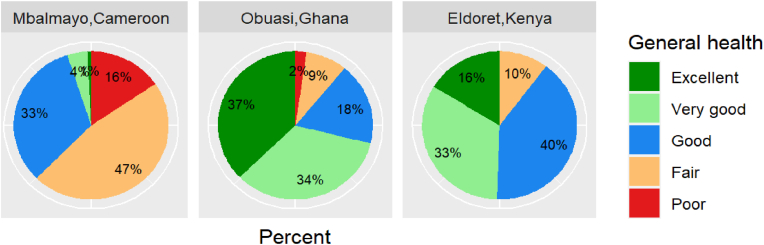
Participant responses to general health question of SF-36 by community.

The first question of the SF-36 asks participants to self-rate their ‘general health’. Responses varied drastically by community; approximately 16% of female Cameroonian participants rated their general health as “poor” compared with 2% of female Ghanaians and no female Kenyans (Fig. 1). Conversely, only 1% of Cameroonians self-rated their health as “excellent” compared with 37% of Ghanaians and 16% of Kenyans.

The average MCS (45.5) and PCS (45.3) scores in Mbalmayo, Cameroon were approximately 5–7 points lower than average MCS and PCS scores in Eldoret, Kenya (49.2 and 51.4, respectively) and Obuasi, Ghana (51.5 and 52.4, respectively) (Table 1). Compared with Eldoret and Mbalmayo, participants in Obuasi had higher average scores across nearly all SF-36 domains (Table 1). Notably, the average general health domain score among participants in Mbalmayo (50.9) was 22 points lower than that in Eldoret (73.1), and 32 points lower than the average in Obuasi (82.7) (Table 2). Further, average physical health and physical pain domain scores in Mbalmayo (66.0 and 58.0, respectively) were approximately 15–25 points lower than the reported averages in Obuasi (90.8 and 73.9, respectively) and Eldoret (80.6 and 82.9, respectively). The average emotional role functioning domain score was also 15–20 points lower in Mbalmayo (69.8) compared with Eldoret (83.0) and Obuasi (90.6).

**Table 2:** Mean (SD) of SF-36 domains and summary scores of female participants by community.

	Overall (N ​= ​1,150)	Mbalmayo, Cameroon (N ​= ​399)	Obuasi, Ghana (N ​= ​346)	Eldoret, Kenya (N ​= ​405)
**Mental component summary (MCS) score**	48.6 (8.6)	45.5 (9.1)	51.5 (7.0)	49.2 (8.1)
**Physical component summary (PCS) score**	49.6 (8.9)	45.3 (9.2)	52.4 (7.1)	51.4 (8.5)
**General health**	68.2 (21.6)	50.9 (16.0)	82.7 (18.8)	73.1 (16.5)
**Physical health**	78.6 (37.3)	66.0 (43.7)	90.8 (22.8)	80.6 (36.7)
**Physical role function**	87.5 (20.2)	85.7 (21.9)	88.6 (17.3)	88.6 (20.6)
**Physical pain**	71.5 (26.8)	58.0 (27.2)	73.9 (21.1)	82.9 (24.6)
**Vitality (energy)**	61.3 (17.4)	58.8 (16.3)	70.6 (18.7)	55.8 (14.0)
**Social function**	77.6 (25.0)	67.7 (25.9)	83.5 (21.9)	82.3 (23.5)
**Emotional role function**	80.3 (36.0)	69.0 (41.1)	90.3 (25.8)	82.8 (34.8)
**Mental health**	72.5 (16.0)	66.7 (15.6)	74.7 (15.6)	76.5 (15.8)

### 3.2 Estimating the prevalence of depression

**Fig. 2:**
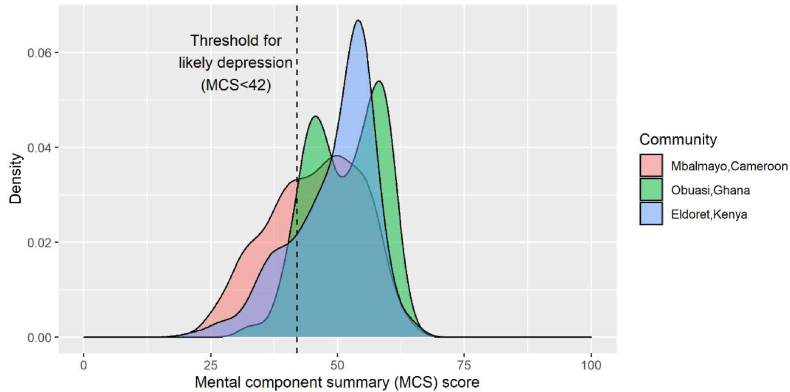
Distribution of MCS scores by community.

Approximately 22% (n ​= ​252) of female participants met the criteria for likely depression using the established cut-off point for MCS score (MCS<42%) (Fig. 2) (Silveira et al., 2005). The prevalence of depression varied four-fold between communities; 36% (n ​= ​143) of female participants in Mbalmayo, 9% (n ​= ​30) in Obuasi and 20% (n ​= ​79) in Eldoret were likely depressed (Table 3). In Eldoret, the prevalence of likely depression was twice as high among female participants using a polluting primary cooking fuel (32%; n ​= ​53) compared with females cooking mostly with LPG (16%; n ​= ​26) (Table 3). Using the mental health domain score cut-off (<52), the prevalence of likely depression was consistently 50–75% lower than the MCS score cut-off in all communities (Table 3). Among males, the prevalence of likely depression was higher than that of females (59%; n ​= ​13) in Mbalmayo, but lower in Obuasi (0%) and Eldoret (13%; n ​= ​3).

**Table 3:** Estimated prevalence of depression among female participants using proposed SF-36 score thresholds (Silveira et al., 2005) (Proportion (n)).

Characteristic	Prevalence of depression using mental component summary (MCS) score threshold (42)			Prevalence of depression using mental health domain score threshold (52)		
	Mbalmayo, Cameroon (n ​= ​404)	Obuasi, Ghana (n ​= ​348)	Eldoret, Kenya (n ​= ​405)	Mbalmayo, Cameroon (n ​= ​404)	Obuasi, Ghana (n ​= ​348)	Eldoret, Kenya (n ​= ​405)
Overall	36% (143)	9% (30)	20% (79)	16% (64)	3% (9)	5% (21)
Primary cooking fuel						
LPG	37% (70)	7% (11)	16% (26)	20% (35)	1% (2)	2% (3)
Polluting∗	33% (73)	11% (19)	32% (53)	13% (29)	4% (7)	8% (18)
Electricity access						
Yes	22% (90)	9% (2)	18% (30)	3% (9)	8% (19)	5% (13)
No	55% (53)	9% (28)	23% (49)	0	27% (45)	6% (8)
# burns while cooking						
0	24% (74)	8% (28)	19% (73)	10% (32)	2% (8)	5% (20)
1	27% (5)	29% (2)	0	27% (5)	0	0
2+	86% (64)	0	50% (6)	36% (27)	1 (50%)	8% (1)

### 3.3 SF-36 descriptive results by energy poverty indicators

When comparing SF-36 results by primary cooking fuel type across all communities, average MCS and PCS scores were 1.3 and 2.4 points higher, respectively among female participants primarily cooking with LPG compared to those primarily using polluting fuels (Table 2). However, the difference in average MCS score and PCS score between LPG versus polluting cooking fuel groups varied by community (Table 4). Among males, those primarily cooking with LPG had average MCS and PCS scores that were 3.3 and 2.5 points higher, respectively, than those primarily cooking with polluting fuels (Table 5).

**Table 4:** Mean (SD) of SF-36 domains and summary scores among female participants by primary cooking fuel type and community.

	Overall (N ​= ​1,150)		Mbalmayo, Cameroon (N ​= ​399)		Obuasi, Ghana (N ​= ​346)		Eldoret, Kenya (N ​= ​405)	
Primary cooking fuel	LPG (N ​= ​527)	Biomass (N ​= ​623)	LPG (N ​= ​177)	Biomass (N ​= ​222)	LPG (N ​= ​166)	Biomass (N ​= ​180)	LPG (N ​= ​184)	Biomass (N ​= ​221)
**Mental component summary (MCS) score**	49.3 (8.2)	48.0 (8.8)	45.1 (8.9)	45.8 (9.3)	52.5 (6.8)	50.6 (7.2)	50.4 (6.9)	48.2 (8.9)
**Physical component summary (PCS) score**	50.9 (8.0)	48.5 (9.5)	46.4 (8.3)	44.5 (9.8)	53.1 (6.3)	51.7 (7.7)	53.2 (7.2)	49.9 (9.1)
**General health**	71.1 (20.8)	65.8 (22.0)	53.1 (15.0)	49.1 (16.5)	85.0 (17.0)	80.6 (20.1)	76.0 (15.5)	70.6 (16.8)
**Physical health**	82.8 (33.4)	74.9 (40.0)	68.5 (41.9)	64.1 (45.1)	92.1 (19.1)	89.6 (25.8)	88.5 (29.6)	74.0 (40.6)
**Physical role functioning**	90.0 (17.9)	85.5 (21.8)	89.0 (19.8)	82.9 (23.3)	90.0 (14.4)	87.3 (19.6)	90.8 (18.6)	86.7 (21.9)
**Physical pain**	74.3 (26.7)	69.2 (26.7)	58.0 (27.9)	58.1 (26.6)	76.3 (20.3)	71.7 (21.6)	88.4 (20.9)	78.4 (26.5)
**Vitality (energy)**	63.0 (17.3)	59.8 (17.3)	58.6 (15.4)	58.9 (17.0)	73.0 (17.8)	68.3 (19.3)	58.2 (15.0)	53.8 (12.6)
**Social functioning**	80.4 (26.1)	75.2 (23.5)	68.4 (24.6)	67.2 (27.0)	86.5 (20.2)	80.7 (23.1)	86.3 (20.0)	78.8 (25.5)
**Emotional role functioning**	84.0 (32.2)	77.1 (38.5)	72.4 (39.2)	66.4 (42.4)	91.3 (23.0)	89.4 (28.2)	88.6 (28.8)	78.0 (38.4)
**Mental health**	73.1 (16.1)	72.0 (15.9)	65.0 (15.6)	68.0 (15.4)	76.5 (14.6)	73.1 (14.4)	78.0 (14.0)	75.1 (16.9)

**Table 5:** Mean (SD) of SF-36 domain and summary scores among male participants by primary cooking fuel type and community.

	Overall (N ​= ​58)		Mbalmayo, Cameroon (N ​= ​24)		Obuasi, Ghana (N ​= ​10)		Eldoret, Kenya (N ​= ​24)	
Primary cooking fuel	LPG (N ​= ​41)	Biomass (N ​= ​17)	LPG (N ​= ​16)	Biomass (N ​= ​8)	LPG (N ​= ​7)	Biomass (N ​= ​3)	LPG (N ​= ​18)	Biomass (N ​= ​6)
**Mental health component summary (MCS) score**	48.9 (8.3)	45.6 (10.8)	41.8 (5.8)	39.7 (10.3)	53.4 (5.8)	52.0 (7.0)	53.0 (7.0)	49.1 (10.8)
**Physical health component summary (PCS) score**	53.2 (7.3)	50.7 (8.8)	48.8 (8.5)	50.1 (10.3)	56.4 (2.0)	48.6 (10.0)	55.6 (6.0)	52.6 (7.7)
**General health**	75.4 (20.0)	67.4 (20.7)	55.6 (8.3)	53.1 (13.3)	94.3 (4.5)	76.7 (28.4)	85.6 (15.8)	81.7 (13.1)
**Physical health**	89.0 (30.2)	73.5 (42.8)	85.9 (41.0)	78.1 (34.1)	96.4 (9.5)	75.0 (43.3)	88.8 (32.3)	66.7 (51.6)
**Physical role functioning**	95.0 (17.2)	90.1 (20.6)	89.4 (26.6)	88.8 (24.3)	100 (0)	78.3 (29.3)	98.3 (4.9)	97.5 (6.2)
**Physical pain**	75.7 (26.9)	76.3 (25.7)	57.3 (26.6)	72.8 (32.4)	75.7 (18.8)	76.7 (13.5)	92.1 (20.3)	80.8 (21.5)
**Vitality (energy)**	64.3 (17.2)	66.8 (16.6)	52.2 (10.2)	70.0 (19.5)	78.6 (17.3)	68.3 (17.6)	69.4 (15.8)	61.7 (13.3)
**Social functioning**	80.0 (24.2)	69.5 (30.6)	60.8 (20.5)	55.4 (29.6)	96.4 (6.1)	79.2 (26.0)	89.6 (21.1)	81.3 (31.4)
**Emotional role functioning**	92.7 (26.4)	72.6 (41.2)	87.5 (34.2)	54.2 (43.4)	100 (0)	100 (0)	94.4 (23.6)	83.3 (40.8)
**Mental health**	69.8 (18.8)	65.6 (21.0)	53.8 (8.1)	57.0 (21.1)	73.7 (14.4)	69.3 (23.4)	82.4 (16.9)	75.3 (18.0)

The largest discrepancies in average SF-36 domain scores between LPG and polluting cooking fuel groups were physical health (LPG: +7.9 points), emotional role functioning (LPG: +6.9 points), general health (LPG+: 5.3 points) and social functioning (LPG: +5.2 points), although the relative difference between fuel groups varied considerably by community (Table 2). Among male participants, the same four domains had the largest discrepancies in average scores between fuel groups as among females, but the relative difference in scores among males primarily using LPG versus biomass (Table 3) was greater than that of female participants (Table 2). For example, there was an approximately 20-point and 10-point higher average emotional and social role functioning domain scores among males in households cooking primarily with LPG compared with polluting fuels, respectively (Table 3), compared with around an 8-point and 4-point increase in average emotional and social role functioning domain scores, respectively, among female participants. The 20-point higher average emotional role functioning domain score among men primarily cooking with LPG was largely driven by males in Mbalmayo, Cameroon (LPG: +33.3), while the 15-point higher physical health domain scores were mainly due to self-rated responses by males in Eldoret, Kenya (LPG: +22.1) and Obuasi, Ghana (LPG: +21.4).

**Fig. 3:**
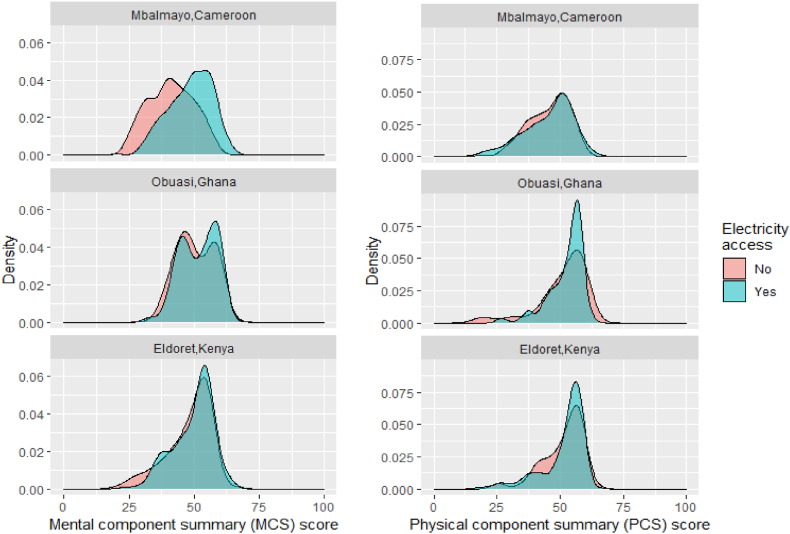
Distribution of mental (MCS) and physical component summary (PCS) scores by community and electricity access.

Having access to electricity for lighting was more strongly associated with increased MCS scores than PCS scores, with the largest effect among participants in Mbalmayo (Fig. 3). In Mbalmayo, the prevalence of likely depression was 2.5 times higher among those without access to electricity (55%) compared to those with access (22%) (Table 3).

**Fig. 4:**
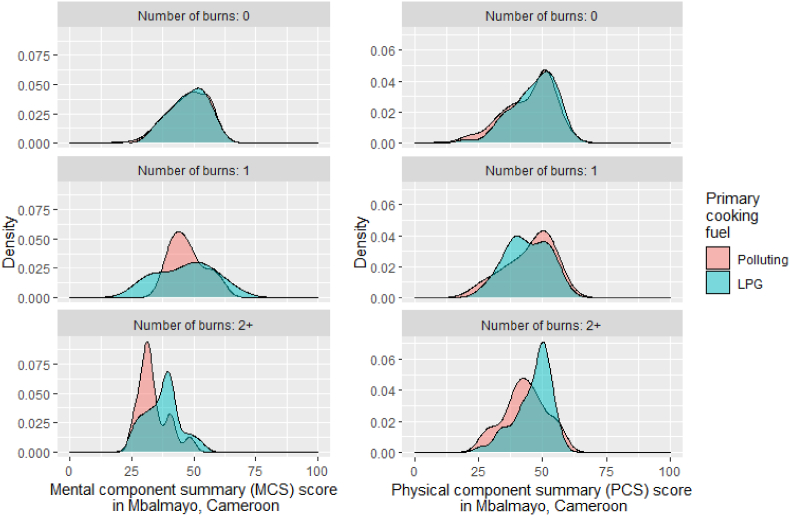
Distribution of mental (MCS) and physical component summary (PCS) scores in Mbalmayo, Cameroon by number of cooking-related burns during the previous year.

Among participants who reported suffering at least two burns during the previous year in Mbalmayo, there was a larger difference in both MCS and PCS scores between those cooking primarily with LPG and those exclusively using polluting fuels, compared to those that had not been burned (Fig. 4).

**Fig. 5:**
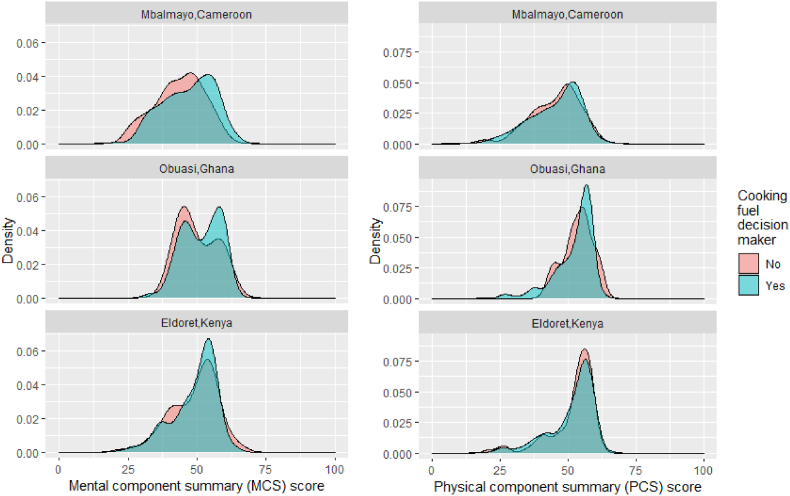
Distribution of mental (MCS) and physical component summary (PCS) scores by community and whether the participant has authority over cooking-related decisions for the household.

Having authority of household cooking-related decisions was more highly positively associated with MCS scores as opposed to PCS scores in Mbalmayo and Obuasi (Fig. 5). In Mbalmayo, female participants in charge of cooking-related decisions had 7-point and 8-point higher social role functioning (Supplementary Fig. 2) and mental health domain scores (Supplementary Fig. 3), respectively.

### 3.4 Mental health-related quality of life modelling

**Fig. 6:**
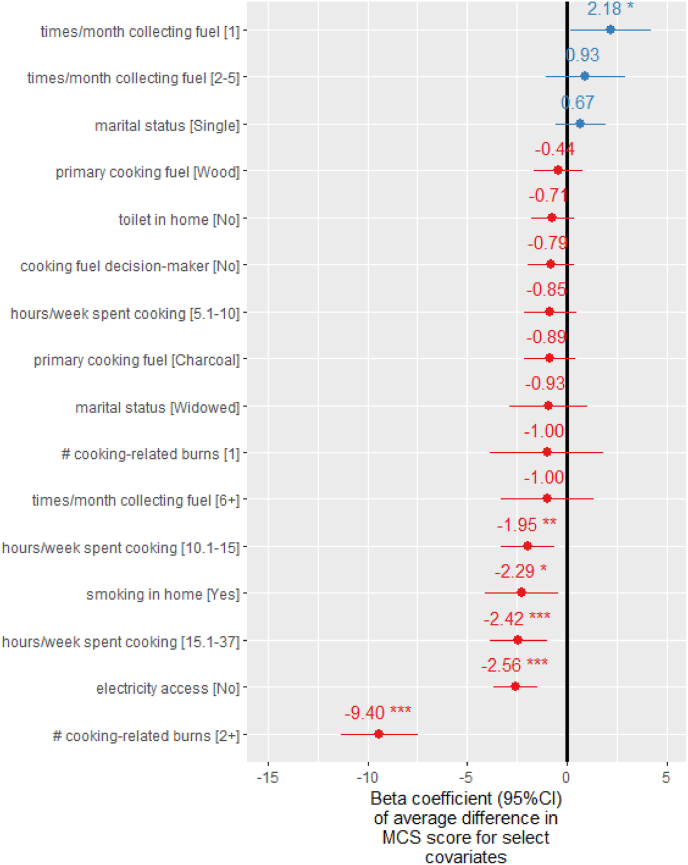
Select Beta coefficients (95% CI) from linear random effects model for mental component summary (MCS) score (interaction effects not shown for simplicity). Full list of Beta coefficients is available in Supplementary Table 4.

The linear random effects model of MCS scores (Supplementary Table 4) moderately explained the variability in the outcome (R^2^_conditional_ ​= ​0.26; R^2^_marginal_ ​= ​0.23; cross validation (CV) R^2^ ​= ​0.23 root mean squared error (RMSE) ​= ​7.07) (Supplementary Table 5). The characteristics significantly negatively associated with mental HRQoL among the female study population were being burned twice or more during the previous year while cooking (β ​= ​−9.4 95%CI:[−11.0,-7.5]), not having access to electricity (β ​= ​−2.6 95%CI:[−3.7, −1.5]) and cooking 10–15 ​h (β ​= ​−1.9 95%CI:[−3.3,-0.6]) or more than 15 ​h per week (−2.4 95% CI:[−3.8, −1.0]), compared with those cooking 0–5 ​h per week (Fig. 6). Females who had no decision authority for choice of cooking fuels (β ​= ​−0.8 95% CI:[−1.9,0.4]) and living in homes with active smokers (β ​= ​−2.3 95%CI:[−4.1,0.5]) also had marginally significantly lower mental HRQoL.

In the linear fixed effects model of 10.13039/100011715MCS scores, participants with higher financial security (enough money to support family needs) in Mbalmayo, Cameroon and Eldoret, Kenya had significantly lower 10.13039/100011715MCS scores than those indicating insufficient financial security (Supplementary Table 4). In the model considering primary and secondary cooking fuel type, households using LPG as a secondary fuel with a polluting primary fuel had lower MCS scores than those exclusively cooking with LPG (Supplementary Table 7); the model fit did not improve when accounting for stove stacking (R^2^_marginal_ ​= ​0.23).

In the Poisson mixed effects regression model (whether the participant is likely depressed based on MCS score <42), there was a monotonically increasing relationship between the prevalence of likely depression and number of cooking-related burns suffered (Supplementary Table 8). Females burned twice or more while cooking in the previous year had 2.7 (95%CI:[1.8,4.1]) times the odds of depression as those not burned (Supplementary Table 8). The positive association was strongest in Mbalmayo, where 86% (n ​= ​64) of females burned twice or more while cooking during the previous year were likely depressed, compared with 24% among those not burned.

Females without electricity access had 1.4 (95%CI:[1.1,2.0]) times the odds of likely depression as those with access. Females primarily cooking with charcoal (1.6 95%CI:[0.9,2.7]) and wood (1.5 95%CI:[0.8,3.0]) had marginally significantly higher odds of likely depression as those primarily cooking with LPG.

### 3.5 Physical health-related quality of life modelling

The linear random effects model of PCS scores performed similarly to the MCS model (R^2^_conditional_ ​= ​0.32; R^2^_marginal_ ​= ​0.21; CV R^2^ ​= ​0.25; RMSE ​= ​7.11) (Supplementary Table 9). However, the most significant predictors of PCS scores (Supplementary Table 11) were different from those of MCS scores, with the exception of being single and spending more hours cooking per week; being single was positively associated with both outcomes, while increased weekly cooking time was associated with a decrease in MCS score but an increase in PCS score. PCS and MCS scores were poorly correlated (R^2^ ​= ​0.13) across all communities (Supplementary Fig. 12).

**Fig. 7:**
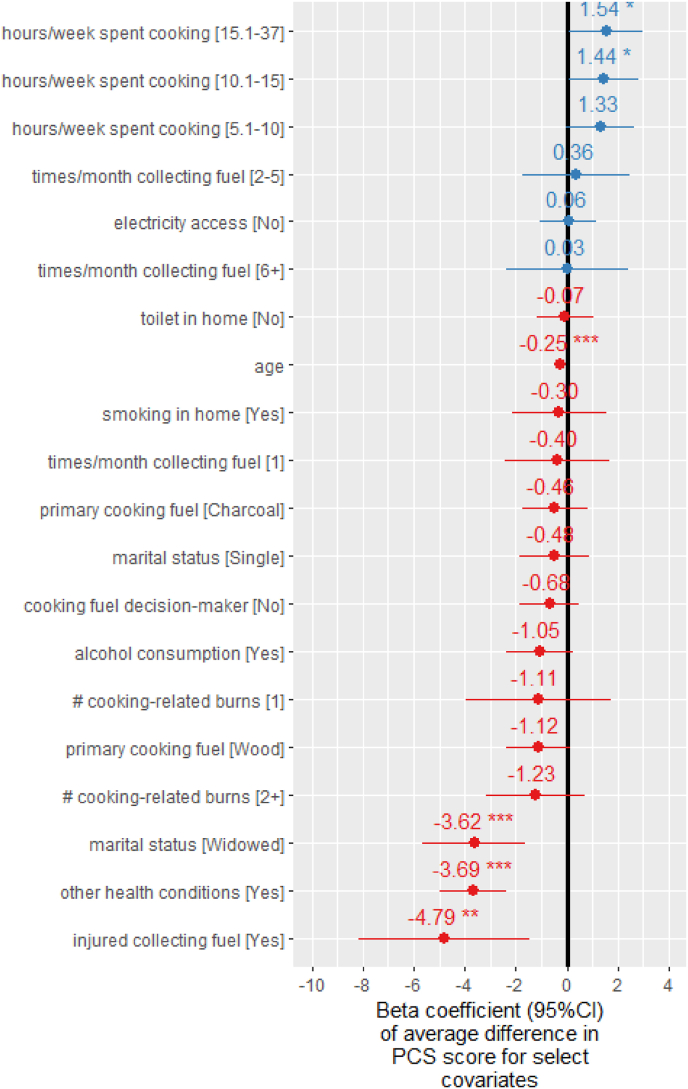
Select Beta coefficients (95% CI) from linear random effects model for physical component summary (PCS) score (interaction effects not shown for simplicity). Full list of Beta coefficients is available in Supplementary Table 11.

Increasing age was significantly negatively associated (β ​= ​−0.25 95%CI:[−0.2,-0.3]) with PCS score (Supplementary Table 11), while it was positively associated with MCS score. Electricity access was not significantly associated with PCS score in the modelling, while being strongly positively associated with mental HRQoL. Although being burned while cooking during the last year was the most important predictor of MCS score, it was not associated with physical HRQoL in the multivariable model. The strongest predictor of PCS score was whether the participant was injured while collecting fuelwood for cooking during the previous year (β ​= ​−4.8 95%CI:[−8.1,-1.4]) (Fig. 7). Having a physical condition (β ​= ​−3.7 95%CI:[−5.0,-2.4]) and being widowed (β ​= ​−3.6 95%CI:[−5.6,-1.6]) were also significantly negatively associated with PCS (Fig. 7). Female participants primarily using wood had a 1.1 point lower (95%CI:[−2.4,0.2])) average PCS score than those primarily cooking with wood (Fig. 7).

## 4 Discussion

This study quantitatively examined the relationship between energy poverty and HRQoL in peri-urban communities in Cameroon, Ghana and Kenya. Multiple indicators of energy poverty, including longer cooking times, lack of access to electricity and being burned while cooking or injured while collecting fuelwood, were significantly associated with lower mental (Fig. 6) or physical HRQoL (Fig. 7) among females. Suffering from multiple cooking-related burns during the previous year was the factor most strongly associated with lower mental HRQoL and significantly higher odds of probable depression; females experiencing two or more cooking-related burns during the previous year had 2.7 (95%CI:[1.8,4.1]) times the odds of likely depression as those experiencing no burns.

While it is possible that supervising children in the cooking area may predispose women to a greater risk of sustaining cooking-related burns (Rybarczyk et al., 2017), we did not find an increased prevalence of burns among women with children under five years old (Supplementary Table 12). Other research conducted in high-income countries suggests that having a mental health disorder predisposes individuals to a greater burn risk because of higher impulsiveness (Wisely et al., 2010; Palmu et al., 2010), females with poorer mental health in peri-urban communities of SSA may be substantially more prone to burn accidents while cooking. The potential of impulsivity being a mechanism by which cooking-related burns and mental well-being are related is supported by the finding of poorer emotional and social role functioning among females experiencing cooking-related burns (Supplementary Fig. 4) and may explain why burn victims did not have significantly lower 10.13039/100015534PCS scores than those not experiencing any burns (Supplementary Table 11). A low burn severity reported by the majority (>90%) of study participants (Supplementary Table 13) may also partially explain the lower association between suffering from a cooking-related burn and PCS scores relative to MCS scores. As approximately one-quarter (24%) of female participants in Mbalmayo reported suffering a cooking-related burn during the previous year, greater access to mental health care among this population may have the co-benefit of lower burn risk from cooking.

Additionally, women (Table 4) and men (Table 5) cooking with LPG and with access to electricity for lighting (Supplementary Figs. 3–4) had higher SF-36 emotional and social role functioning domain scores than those cooking polluting cooking fuels. The differences in emotional/social role functioning domain scores between those using LPG versus polluting fuels were similar to or greater than that of many SF-36 physical health domains. Previous studies have also identified that clean household energy access may offer individuals greater participation in social activities and an improved social image (Lin and Okyere, 2021; Middlemiss et al., 2019; O’ Sullivan and Nriagu, 2019). Consequently, assessing depressive symptoms associated with energy poverty using only the SF-36 mental health domain may lead to an underestimate of its prevalence by not considering emotional and social role functioning. This likely explains the higher estimated proportion of likely depression in the three study communities when using a cutoff based on the MCS score (which factors in the emotional and social role functioning domains) compared with the threshold derived from the mental health domain only (Table 3).

In line with another study conducted in Ghana (Lin and Okyere, 2021), we find that the association between access to clean cooking fuels and HRQoL may depend on household income. A study in Indonesia similarly found that perceived economic status influenced the relationship between air pollution and mental health (Kim et al., 2020), and a study from Ghana found that income mediated the relationship between energy poverty and well-being (Lin and Okyere, 2021). A weaker association between primary cooking fuel type and mental/physical HRQoL among those with lower income may be due to more attention directed to more pressing worries, including chronic stress and increased negative life events associated with poverty (Lund et al., 2010; Hjelm et al., 2017) and food insecurity (Hadley et al., 2008; Sorsdahl et al., 2011; Perkins et al., 2018). It is also possible that economically secure individuals using polluting cooking fuels are more likely to perceive a lack of access to modern cooking fuels to be misaligned with their life expectations (Druic ă et al., 2019).

### 4.1 Time poverty

Increased cooking time was also a significant predictor of both mental (Fig. 6) and physical HRQoL (Fig. 7). Those cooking more than 10 ​h per week had significantly lower MCS scores than those cooking less than 5 ​h across all three peri-urban communities. This finding may be due lower income and greater fatigue associated with time poverty (Breitkreuz et al., 2010), as a systematic review found time savings from cooking with LPG compared to wood led to 0.7 ​h/day in time savings and a corresponding 4% increase in daily income (Simkovich et al., 2019). Moreover, time spent gathering fuel required for cooking can also lead to poorer physical HRQoL (Supplementary Fig. 7) due to risk of injury during cooking fuel collection (bodily pain SF-36 domain) (Supplementary Fig. 8). The decrease in PCS score associated with an injury during fuel collect was greater than that of being diagnosed with a chronic health condition (−3.7 95%CI:[−5.0, −2.3]).

The prevalence of likely depression varied by a factor of four between communities (from 36% in Mbalmayo to 9% in Obuasi based on the MCS score cut-off point) (Table 3). Although the prevalence of depression was lower using the mental health domain score criterion (Table 3), the estimated prevalence of likely depression remained a factor of four times higher in Mbalmayo compared with Obuasi and Eldoret. While some variation between communities may be attributed to cultural differences, higher estimates of depression prevalence in Mbalmayo may partially be due to much lower levels of financial security reported by females compared with those in Obuasi and Eldoret (Table 1). Cameroon is the least economically developed study country in terms of gross domestic product per capita; Cameroon's GDP per capita ($1,499 USD) is lower than that of Kenya ($1,832 USD) and Ghana ($2,329 USD) (World Development Indicators, 2020). Increased country-level economic development, which can foster women's empowerment via several mechanisms (Duflo, 2012), may partly explain the large community-level variation in estimated depression prevalence.

While there was a positive relationship between greater financial security and higher MCS score in Obuasi, Ghana, females reporting financial security in Eldoret and Mbalmayo had significantly lower MCS scores (Supplementary Table 4) than those without financial security. This finding has been echoed by other studies, which found that an association between lower prevalence of mental health disorders with decreasing poverty levels at a country level is not always mirrored at the household level (Lund et al., 2010).

### 4.2 Comparing quality of life by sex and age

Although the sample of male participants was small (n ​= ​58), the four SF-36 domains most affected by energy poverty (general health, physical health, emotional role functioning, social role functioning) were the same between sexes. Hence, energy poverty likely also adversely affects men, despite women mainly conducting the household cooking activities. The mechanisms by which energy poverty impacts life satisfaction may also be similar between sexes. However, a low correlation between MCS and PCS scores among men and women (Supplementary Fig. 9) indicates that the pathways by which energy poverty affects physical and mental HRQoL are distinct.

In contrast to sex, there were substantial differences in HRQoL by age. While older females were more likely to have lower physical HRQoL (Fig. 7), younger individuals (aged 18–26 years) had the lowest mental HRQoL (Fig. 6), indicating a potentially uniquely vulnerable target group for mental health care. Lower MCS scores among young people may reflect struggles to earn a livelihood in highly competitive urbanising labour markets in SSA. Because this competition will intensify due to Africa's growing population, mental health problems among young Africans may increase if they fail to realise their ambitions (Sankoh et al., 2018).

### 4.3 Strengths and limitations

The results from this study importantly highlight how alleviating energy poverty in peri-urban communities in SSA with a primary aim of reducing exposure to air pollution and achieving associated positive climate and physical health impacts may have the potential co-benefit of improved well-being. However, a causal relationship between factors associated with energy poverty and mental well-being cannot be established as this study was cross-sectional. An examination of changes in mental well-being within longitudinal or intervention studies aimed at reducing energy poverty is warranted. This study further demonstrates that evaluating the mental health domain of SF-36 alone as an outcome in energy poverty studies may lead to underreporting of possible depression due to omission of emotional and social functioning measures.

Given the large sample size of this study, the highly significant relationships between multiple SF-36 domains and indicators of energy poverty likely reflect true associations. However, it is possible that omitted variable bias exists in this study as both energy poverty and well-being are complex phenomena influenced by a variety of social, cultural, biological and environmental factors. For example, some factors that may affect energy poverty and HRQoL (e.g. food security, water security) may not have been adequately assessed. Food insecurity has been found to be significantly associated with poorer mental health status (Sweetland et al., 2019) including through its link with an increase in intimate partner violence in peri-urban SSA (Hatcher et al., 2019). We note that women cooking with LPG had significantly lower odds of likely depression than those cooking with charcoal when accounting for primary water source in parsimonious models (Supplementary Tables 14–15). However, given known associations between water insecurity with energy poverty and measures of HRQoL or depression (Stevenson et al., 2012; Stoler et al., 2020; Mushavi et al., 2020), a lack of comprehensive measurements of water insecurity in our study may lead to overestimation of the association between energy poverty and HRQoL; future studies should aim to collect more detailed information on food and water insecurity to possibly improve quantification of the relationship.

BMI, which has previously been found to be associated with HRQoL (Kolotkin and Andersen, 2017), was measured in this study. Having a BMI classified as “overweight” or “obese” was marginally significantly associated with a lower PCS score in the multivariable model (Supplementary Table 17), however there was not a significant change in results from the MCS or PCS models when adding BMI as a covariate (Supplementary Tables 16–17). Questions about intimate partner violence, which can adversely affect quality of life among women (Conroy, 2014; Winter et al., 2020), were not asked in the survey. Regardless of whether autonomy in cooking choices signals a lower risk of experiencing domestic abuse, female cooks in charge of making decisions on household cooking fuels had higher SF-36 mental health domain scores than those who had no decision authority (Supplementary Fig. 3), reinforcing the importance of women's empowerment for improving quality of life.

Due to use of stratified randomization, socioeconomic status of the study communities may have been artificially inflated. However, sensitivity analyses found no significant differences in several key sociodemographic characteristics between the Phase 2 study population and population-based surveys in Phase 1 (Supplementary Table 18). The relationship between energy poverty and HRQoL found among peri-urban communities in this study may not be nationally representative as types of cooking fuels used and other socioeconomic factors that confound the relationship between clean energy access and well-being can vary substantially in rural, urban and peri-urban settings. Additionally, with a focus on access to clean cooking fuels, this study does not include all indicators of energy poverty, which is a multidimensional phenomenon (Sovacool et al., 2012). With many indices developed to comprehensively measure energy poverty (Nussbaumer et al., 2012; Casta ñ o-Rosa et al., 2020), additional studies are warranted that evaluate other dimensions of energy poverty in relation to HRQoL.

This study did not collect data on diagnosis of anxiety/mood disorders. While future SF-36 questionnaire validation among diagnosed depressed individuals in SSA is warranted, the SF-36 MCS score correlates highly with severity of depressive symptoms (IsHak et al., 2011). With insufficient access to mental health services and a stigma associated with mental illness in sub-Saharan Africa (Sankoh et al., 2018), using the SF-36 can possibly help overcome social desirability bias that may lead to underdiagnosis of depression. As the SF-36 is easy to conduct, it can be an effective depression screening tool in resource poor settings.

## 5 Conclusion

With epidemiological research related to energy poverty has primarily focused on physical health impacts, this study generates empirical evidence on additional adverse mental HRQoL effects. The findings suggest that providing universal access to clean cooking fuels (Sustainable Development Goal 7) can plausibly help improve daily emotional and social functioning at a population level. Future epidemiological studies should consider quality-of-life measurements as a means of wholistically evaluating the health burden of cooking with polluting fuels. As the Global Burden of Disease currently only accounts for adverse physical outcomes due to exposure to household air pollution, the overall morbidity due to polluting fuel use may be higher than expected if a lack of access to clean household energy is an independent risk factor for depression. Additional epidemiological studies that examine the manifestation of clinical mental health outcomes in relation to aspects of energy poverty in LMICs can help improve future global risk estimates.

## CRediT authorship contribution statement

**Matthew Shupler:** assisted with the survey design, supervised the data collection, managed, cleaned and analysed all the data, and wrote the first and final drafts of the paper. **Miranda Baame:** led the data collection and study logistics in their respective study countries. **Emily Nix:** assisted with data cleaning, management and interpretation. **Theresa Tawiah:** led the data collection and study logistics in their respective study countries. **Federico Lorenzetti:** assisted with data cleaning, management and interpretation. **Jason Saah:** led the data collection and study logistics in their respective study countries. **Edna Sang:** led the data collection and study logistics in their respective study countries. **Elisa Puzzolo:** assisted with the survey design, designed and supervised the conduct of the CLEAN-Air(Africa) study. helped oversee the data management in the CLEAN-Air(Africa) study. **Judith Mangeni:** led the data collection and study logistics in their respective study countries. **Emmanuel Betang:** led the data collection and study logistics in their respective study countries. **Mieks Twumasi:** helped oversee the data management in the CLEAN-Air(Africa) study. **Seeba Amenga-Etego:** helped oversee the data management in the CLEAN-Air(Africa) study. All the co-authors reviewed and commented on the final manuscript. **Reginald Quansah:** supervised the conduct of the CLEAN-Air(Africa) study and supervised the data collection in their respective countries. **Bertrand Mbatchou:** supervised the conduct of the CLEAN-Air(Africa) study and supervised the data collection in their respective countries. **Diana Menya:** supervised the conduct of the CLEAN-Air(Africa) study and supervised the data collection in their respective countries. **Kwaku Poku Asante:** supervised the conduct of the CLEAN-Air(Africa) study and supervised the data collection in their respective countries. **Daniel Pope:** assisted with the survey design, designed and supervised the conduct of the CLEAN-Air(Africa) study. helped oversee the data management in the CLEAN-Air(Africa) study.

## Declaration of competing interest

The authors have no competing interest to declare.
