# Supplementary material for: Multiple aspects of energy poverty are associated with lower mental health-related quality of life: A modelling study in three peri-urban African communities
Source: SSM Ment Health. 2022 Dec;2:100103. doi: 10.1016/j.ssmmh.2022.100103 (PMC9792378; doi:10.1016/j.ssmmh.2022.100103)
Supplement: Multimedia component 1 [file mmc1.docx]

**Supplementary Information**

**Multiple aspects of energy poverty are associated with lower mental health-related quality of life: A modelling study in three peri-urban African communities**

Matthew Shupler^1*^, Miranda Esong^2^, Emily Nix^1^, Theresa Tawiah^3^, Federico Lorenzetti^1^, Jason Saah^3^, Rachel Anderson de Cuevas^1^, Edna Sang^4^, Elisa Puzzolo^1.5^, Judith Mangeni^4^, Emmanuel Betang^2^, Mieks Twumasi^3^, Seeba Amenga-Etego^3^, Reginald Quansah,^6^ Bertrand Mbatchou^2^, Diana Menya^4^, Kwaku Poku Asante^3,7^, Daniel Pope^1,7^

1. Department of Public Health, Policy and Systems, University of Liverpool, Liverpool, United Kingdom

2. Douala General Hospital, Douala, Cameroon

3. Kintampo Health Research Centre, Research and Development Division, Ghana Health Service, Kintampo North Municipality, Ghana

4. School of Public Health, Moi University, Eldoret, Kenya

5. Global LPG Partnership (GLPGP), 654 Madison Avenue, New York, United States

6. School of Public Health, University of Ghana, Ghana

7. Co-last authors

*Corresponding author: [m.shupler@liverpool.ac.uk](mailto:m.shupler@liverpool.ac.uk)

**Supplementary Figure 1.** SF-36 questions grouped by domain (scale) and summary measure (re-copied from SF-36 user manual)


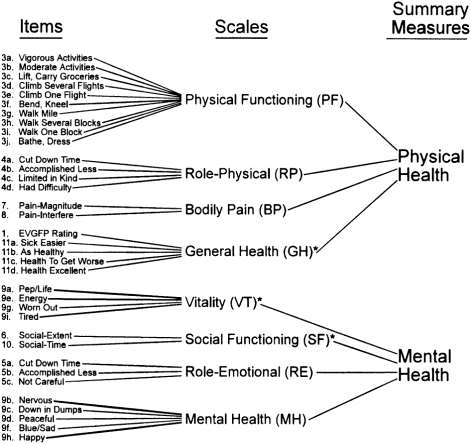


**Supplementary Table 1. Scoring procedure for each question on the SF-36** (questions corresponding to each question number can be obtained here: <https://www.rand.org/health-care/surveys_tools/mos/36-item-short-form/survey-instrument.html>)

| **Question number** | **Response value** | **Score (%):** |
| --- | --- | --- |
| 1, 2, 20, 22, 34, 36 | 1 → | 100 |
|  | 2 → | 75 |
|  | 3 → | 50 |
|  | 4 → | 25 |
|  | 5 → | 0 |
| 3, 4, 5, 6, 7, 8, 9, 10, 11, 12 | 1 → | 0 |
|  | 2 → | 50 |
|  | 3 → | 100 |
| 13, 14, 15, 16, 17, 18, 19 | 1 → | 0 |
|  | 2 → | 100 |
| 21, 23, 26, 27, 30 | 1 → | 100 |
|  | 2 → | 80 |
|  | 3 → | 60 |
|  | 4 → | 40 |
|  | 5 → | 20 |
|  | 6 → | 0 |
| 24, 25, 28, 29, 31 | 1 → | 0 |
|  | 2 → | 20 |
|  | 3 → | 40 |
|  | 4 → | 60 |
|  | 5 → | 80 |
|  | 6 → | 100 |
| 32, 33, 35 | 1 → | 0 |
|  | 2 → | 25 |
|  | 3 → | 50 |
|  | 4 → | 75 |
|  | 5 → | 100 |

**Supplementary Table 2.** Eight domains that each question on SF-36 belongs to (questions corresponding to each question number can be obtained here: <https://www.rand.org/health-care/surveys_tools/mos/36-item-short-form/survey-instrument.html>)

| **Domain** | **Number of items** | **Question numbers** |
| --- | --- | --- |
| Physical functioning | 10 | 3 4 5 6 7 8 9 10 11 12 |
| Physical role functioning | 4 | 13 14 15 16 |
| Emotional role functioning | 3 | 17 18 19 |
| Vitality | 4 | 23 27 29 31 |
| Emotional well-being | 5 | 24 25 26 28 30 |
| Social functioning | 2 | 20 32 |
| Bodily pain | 2 | 21 22 |
| General health | 5 | 1 33 34 35 36 |

**Supplementary Table 3**. Description of variables used in modelling

| **Type** | **Variable** | **Survey question or description** | **Categories** |
| --- | --- | --- | --- |
| Socio-demo-graphic | Age | Participant age | Continuous variable |
|  | Financial security | *Do you feel you have enough money available for your required weekly spending?* | Enough money to support family, not quite enough, definitely not enough |
|  | Education | Highest level of schooling | No formal education, primary school, secondary/high school, university |
|  | Marital status | Marital status | Married, single, widowed/divorced, living with partner |
|  | Head of household | *Are you the head of household?* | Yes, no |
|  | Number of household members | *How many individuals live in the household (including participant)?* | Continuous variable |
|  | Number of rooms in household | *How many rooms (excluding the kitchen and storage room(s)) does this household have?* | Continuous variable |
|  | Landowner | Owns land | Yes, no |
|  | Own a bike | Owns a bike | Yes, no |
|  | Own a car | Owns a car | Yes, no |
|  | Own a television | Owns a television | Yes, no |
| Health-related | Smokers in household | *Does anyone else in your household smoke cigarettes?* | Yes, no |
|  | Physical health condition | *Have you ever been told by a doctor that you have/had any of the following diseases? Tuberculosis, chronic bronchitis, heart disease, high blood pressure* | Yes (if at least one), no (if none) |
|  | Alcohol consumption | *Have you consumed an alcoholic drink such as beer, wine, spirits, fermented drink in the past 12 months?* | Yes, no |
|  | Body mass index (sensitivity analysis only) | Height and weight of every participant were measured at time of the survey | Underweight (<18.5), normal weight (18.5-24.9), overweight (25-29.9), obese (>29.9) |
| Energy poverty | Primary cooking fuel type | *What does this household use for cooking most of the time, including cooking food, making tea/coffee, boiling drinking water?* | LPG, wood, charcoal |
|  | Secondary fuel type (sensitivity analysis only) | *What other fuels does this household use for cooking most of the time, including cooking food, making tea/coffee, boiling drinking water?* | (1) LPG primary, (2) charcoal primary (polluting fuels exclusively), (3) wood primary (polluting fuels exclusively), (4) polluting (wood or charcoal) primary and LPG secondary |
|  | Cooking fuel decision-maker | *Who in your household makes the decision on what fuel is used/ purchased for cooking?* | Myself, partner, other |
|  | Electricity access | *Do you have an electricity connection?* | Yes, no |
|  | Secondary cooking fuel type | *Excluding the stove previously mentioned, what does this household use for cooking most of the time, including cooking food, making tea/coffee, boiling drinking water?* | LPG, wood, charcoal |
|  | Fuel collection per month | *Do you obtain any cooking fuel for free? And In a typical month, on how many occasions would these fuels be collected?* | 0, 1, 2-5, 6+ |
|  | Number of cooking-related burns in last year | *Have you suffered any burns or scalds in the last 12 months? And How many times in the last 12 months?* | 0, 1, 2+ |
|  | Injury while collecting fuel | *In the past year, did you experience an injury while collecting or transporting fuel?* | Yes, no |
| Water & sanitation | Water source | *Where do you obtain most of your water for your household needs?* | From river/rainwater, communal standpipe, pipe in home, pump (deep well), pit with bucket |
|  | Sanitation | *Do you have a septic tank or flushing toilet inside your house?* | Yes, no |

A total of 166 (14%) female participants were diagnosed with at least one physical health condition associated with HAP: tuberculosis, chronic bronchitis, heart disease or hypertension (Supplementary Table 3). Two-thirds of female participants in Mbalmayo consumed alcohol compared with less than 5% in Obuasi and Eldoret (Supplementary Table 2).

***Cooking-related decision maker in Mbalmayo***

**Supplementary Figure 2**. Mean social role functioning domain score among female participants by whether they oversee cooking-relation decisions for the household


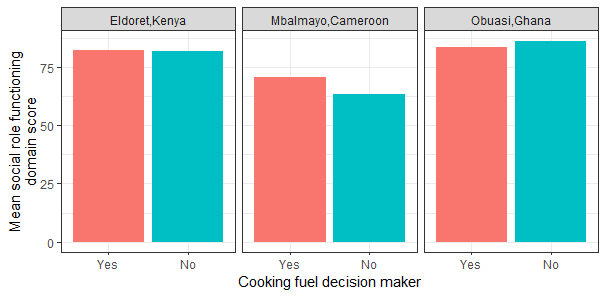


**Supplementary Figure 3**. Mean mental health domain score among female participants by whether they oversee cooking-relation decisions for the household


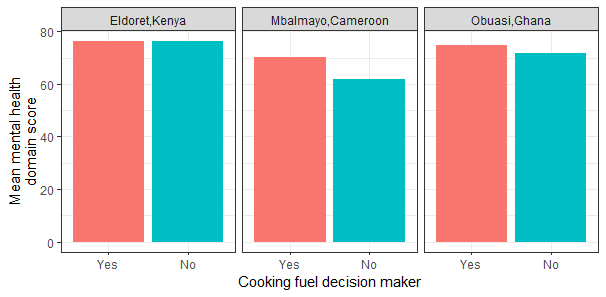


**Supplementary Figure 4.** Mean emotional role functioning domain score (top) and social role functioning domain score (bottom) among female participants by primary cooking fuel type and number of cooking-related burns experienced in the previous year


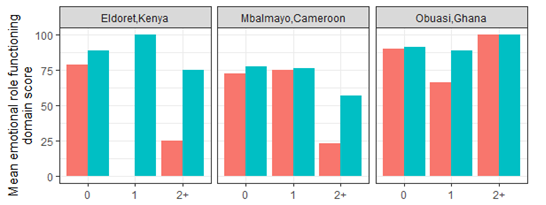


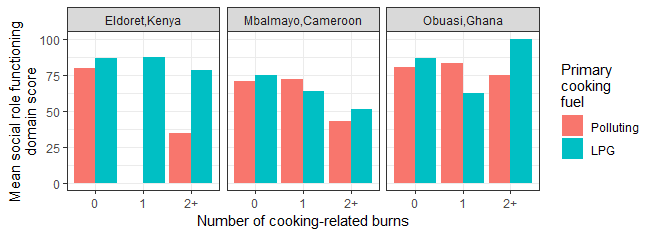


**Supplementary Figure 5**. Mean social role functioning domain score among female participants by community and whether they have access to electricity for lighting


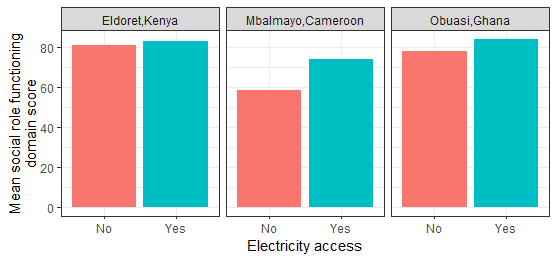


**Supplementary Figure 6**. Mean social role functioning domain score among female participants by community whether they have access to electricity for lighting


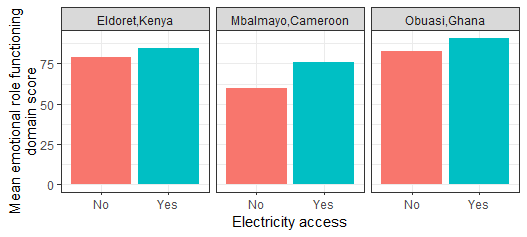


**Supplementary Figure 7**. Distribution of mental (MCS) and physical component summary (PCS) scores by community and suffering an injury during fuelwood collection during the previous year
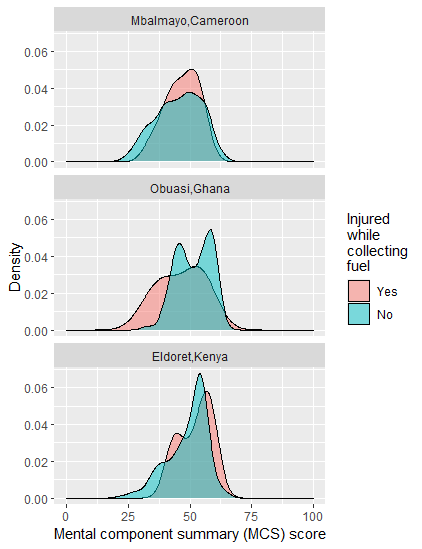

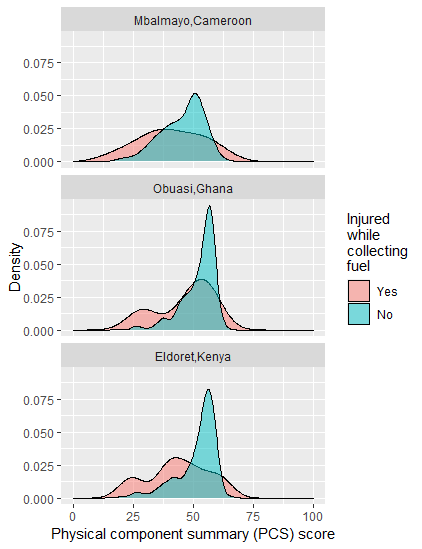


Being injured while collecting fuelwood was more highly negatively associated with physical than mental HRQoL, particularly in Eldoret and Mbalmayo (Supplementary Figure 5).

**Supplementary Figure 8**. Mean bodily pain domain score among female participants by primary cooking fuel type and whether they were injured while collecting cooking fuels during the previous year


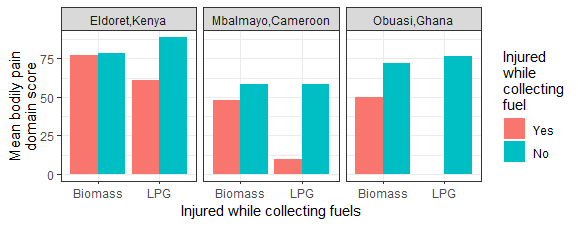


***Relationship between self-perceived mental and physical health***

MCS and PCS scores were poorly correlated (r=0.13) (Supplementary Figure 12).

**Supplementary Figure 9.** Correlation between physical component summary (PCS) and mental component summary (MCS) score


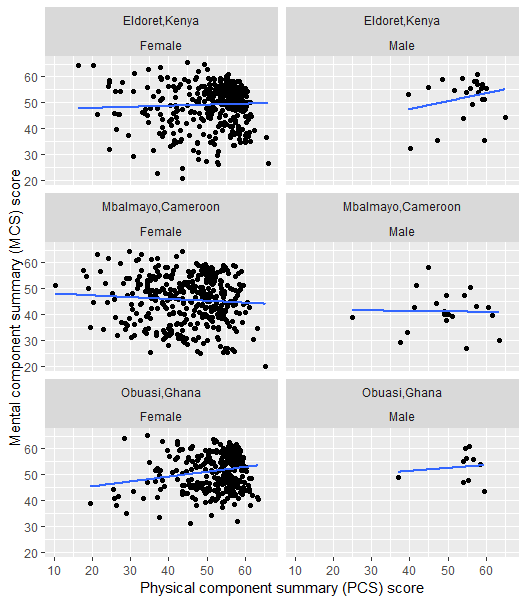


**Supplementary Figure 10**. Comparison of self-reported household income to self-reported financial security


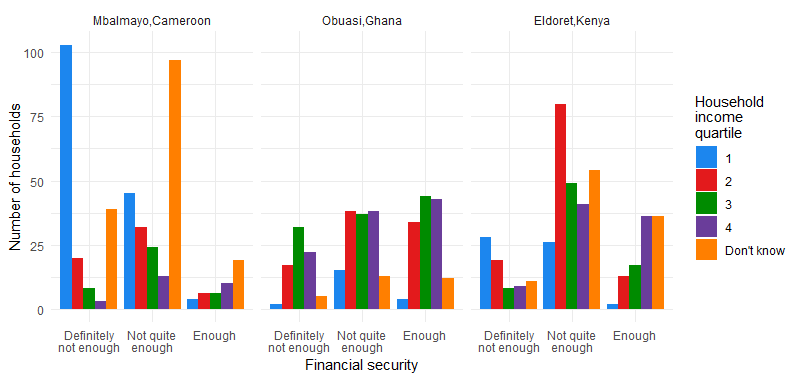


***SF-36 results among female participants***

**Supplementary Figure 11**. Distribution of mental component summary (MCS) and physical component summary (PCS) scores among female participants by primary cooking fuel type and community.


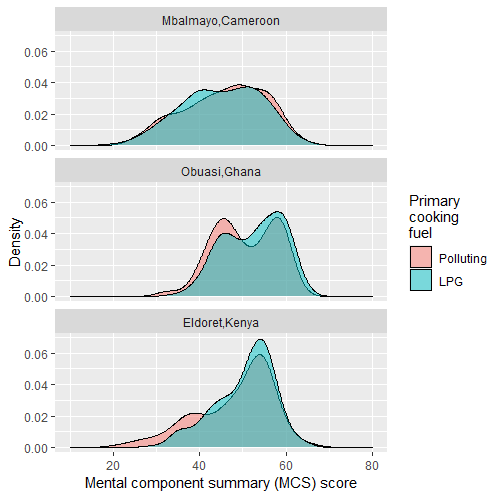

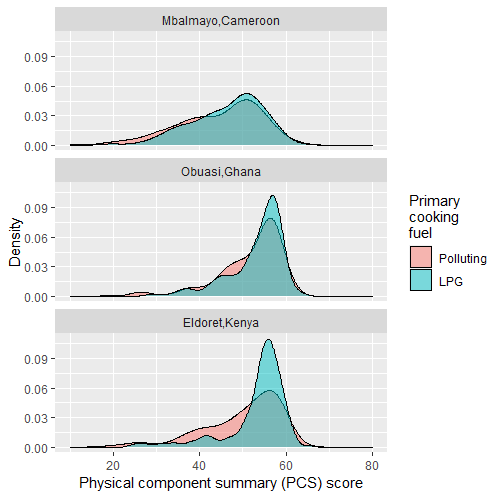


***Emotional and social role functioning scores among burn victims***

Average emotional role functioning (ER) was the SF-36 domain most negatively impacted when individuals reported being burned during the previous year. In Mbalmayo and Eldoret, the average ER score among participants cooking primarily with LPG and experiencing no burns in the last year were roughly 5-10 points higher than those cooking mainly with polluting fuels; however, the average ER score among participants cooking with LPG and experiencing two or more burns in the last year were 35-50 points higher than those cooking mainly with polluting fuels (Supplementary Figure 10). A similar pattern existed among average social role functioning domain scores in Mbalmayo and Eldoret (Supplementary Figure 10).

***Modelling Output***

**Supplementary Table 4.** Coefficients from linear regression of mental component summary (MCS) score (N=1,150)

|  | **Fixed effects model** | | | **Random effects model** | |
| --- | --- | --- | --- | --- | --- |
| **Characteristic** | **Beta** | **95% CI^1^** | **p-value** | **Beta** | **95% CI^1^** |
| Primary cooking fuel |  |  |  |  |  |
| LPG | — | — |  | — | — |
| Charcoal | -1.0 | -2.3, 0.25 | 0.11 | -0.89 | -2.2, 0.40 |
| Wood | -0.74 | -2.0, 0.51 | 0.2 | -0.43 | -1.7, 0.82 |
| Electricity access |  |  |  |  |  |
| Yes | — | — |  | — | — |
| No | -2.6 | -3.7, -1.5 | <0.001 | -2.6 | -3.7, -1.5 |
| Number of fuel collections per month |  |  |  |  |  |
| 0 | — | — |  | **—** | — |
| 1 | 2.0 | 0.01, 4.0 | 0.049 | 2.2 | 0.17, 4.2 |
| 2-5 | 1.0 | -0.95, 3.0 | 0.3 | 0.93 | -1.1, 2.9 |
| 6+ | -0.84 | -3.1, 1.5 | 0.5 | -1.0 | -3.3, 1.3 |
| Cooking fuel decision maker |  |  |  |  |  |
| Yes | — | — |  | **—** | — |
| No | -0.86 | -2.0, 0.28 | 0.14 | -0.79 | -1.9, 0.36 |
| Number of burns |  |  |  |  |  |
| 0 | — | — |  | — | — |
| 1 | -0.72 | -3.5, 2.1 | 0.6 | -1.0 | -3.8, 1.8 |
| 2+ | -9.5 | -11, -7.6 | <0.001 | -9.4 | -11, -7.5 |
| Hours cooking per week |  |  |  |  |  |
| 0-5 | — | — |  | — | — |
| 5.1-10 | -1.1 | -2.4, 0.18 | 0.089 | -0.85 | -2.2, 0.47 |
| 10.1-15 | -2.4 | -3.7, -1.0 | <0.001 | -1.9 | -3.3, -0.61 |
| 15.1-37 | -2.6 | -4.0, -1.2 | <0.001 | -2.4 | -3.8, -1.0 |
| Age | -0.01 | -0.06, 0.04 | 0.6 | 0.00 | -0.05, 0.05 |
| Martial status |  |  |  |  |  |
| Married | — | — |  | — | — |
| Living with partner | 0.18 | -1.3, 1.7 | 0.8 | -0.06 | -1.6, 1.5 |
| Single | 0.80 | -0.55, 2.1 | 0.2 | 0.64 | -0.72, 2.0 |
| Widowed | -0.74 | -2.7, 1.2 | 0.5 | -0.92 | -2.9, 1.1 |
| Number of household members | -0.15 | -0.34, 0.04 | 0.12 | -0.17 | -0.36, 0.02 |
| Household head |  |  |  |  |  |
| Yes | — | — |  | — | — |
| No | 1.4 | 0.06, 2.8 | 0.040 | 1.3 | -0.05, 2.7 |
| Number of rooms | 0.20 | -0.09, 0.50 | 0.2 | 0.12 | -0.18, 0.42 |
| Education |  |  |  |  |  |
| No education | — | — |  | — | — |
| Primary | -3.0 | -5.2, -0.88 | 0.006 | -0.43 | -4.5, 3.7 |
| Secondary | -2.0 | -4.1, 0.11 | 0.063 | 1.1 | -3.0, 5.2 |
| University | -1.8 | -4.2, 0.56 | 0.13 | 3.3 | -1.5, 8.0 |
| Financial security |  |  |  |  |  |
| Definitely not enough | — | — |  | — | — |
| Not quite enough | 4.3 | 2.2, 6.4 | <0.001 | 4.6 | -0.23, 9.4 |
| Enough to buy everything | 7.3 | 5.1, 9.4 | <0.001 | 5.0 | -0.23, 10 |
| Landowner |  |  |  |  |  |
| Yes | — | — |  | — | — |
| No | -0.17 | -1.2, 0.88 | 0.8 | 0.08 | -1.0, 1.1 |
| Smoking inside household |  |  |  |  |  |
| No | — | — |  | — | — |
| Yes | 0.32 | -0.13, 0.73 |  | -2.3 | -4.1, -0.45 |
| Toilet in household |  |  |  |  |  |
| No | — | — |  |  |  |
| Yes | 0.64 | -0.44, 1.7 | 0.2 | 0.71 | -0.38, 1.8 |
| Water source |  |  |  |  |  |
| Collect from river | — | — |  | — | — |
| Communal standpipe | -2.2 | -4.9, 0.59 | 0.12 | -2.4 | -5.2, 0.40 |
| Pipe in home | -2.0 | -4.6, 0.68 | 0.15 | -2.1 | -4.8, 0.54 |
| Pump (deep well) | -2.2 | -5.3, 0.91 | 0.2 | -2.5 | -5.7, 0.60 |
| Well (pit with bucket) | -0.81 | -3.4, 1.7 | 0.5 | -1.0 | -3.6, 1.6 |
| Country * Financial security |  |  |  |  |  |
| Not quite enough * Cameroon | -5.1 | -7.7, -2.4 | <0.001 | N/A |  |
| Enough to buy everything * Cameroon | -8.4 | -12, -5.0 | <0.001 | N/A |  |
| Not quite enough * Kenya | -7.0 | -9.8, -4.1 | <0.001 | N/A |  |
| Enough to buy everything * Kenya | -9.9 | -13, -6.8 | <0.001 | N/A |  |
| Education * Financial security |  |  |  |  |  |
| Primary * Not quite enough | N/A |  |  | -3.6 | -8.8, 1.5 |
| Secondary * Not quite enough | N/A |  |  | -5.0 | -10, -0.03 |
| University * Not quite enough | N/A |  |  | -6.6 | -12, -1.0 |
| Primary * Enough to buy everything | N/A |  |  | -3.1 | -8.9, 2.7 |
| Secondary * Enough to buy everything | N/A |  |  | -2.5 | -8.0, 3.0 |
| University * Enough to buy everything | N/A |  |  | -7.6 | -14, -1.4 |
| Country |  |  |  |  |  |
| Ghana | — | — |  | N/A |  |
| Cameroon | 2.1 | -0.27, 4.4 | 0.083 | N/A |  |
| Kenya | 4.1 | 1.6, 6.6 | 0.001 | N/A |  |

**Supplementary Table 5.** Model performance parameters for linear random effects model of MCS scores

| **#** | **Model** | **Conditional R^2 a^** | **Marginal R^2 b^** | **Change in Marginal R^2^** | **ICC^c^** | **AIC^d^** |
| --- | --- | --- | --- | --- | --- | --- |
| 1 | Base random effects model^1^ | 0.12 | 0.00 | -- | 0.12 | 8118 |
| 2 | Base + primary cooking fuel | 0.13 | 0.01 | 0.01 | 0.12 | 8115 |
| 3 | Base + primary cooking fuel + water source | 0.14 | 0.02 | 0.01 | 0.12 | 8103 |
| 4 | Base + primary cooking fuel + water source + sanitation | 0.15 | 0.03 | 0.01 | 0.13 | 8088 |
| 5 | Base + water source + sanitation + energy poverty indicators^2^ | 0.22 | 0.18 | 0.16 | 0.06 | 7912 |
| 6 | Base + water source + sanitation + energy poverty indicators + demographic variables^3^ | 0.24 | 0.20 | 0.03 | 0.04 | 7904 |
| 7 | Base + water source + sanitation + energy poverty indicators + demographic variables + socioeconomic variables^4^ | 0.25 | 0.22 | 0.02 | 0.04 | 7886 |
| 8 | Base + water source + sanitation + energy poverty indicators + socioeconomic variables + demographic variables + smoking in the household | 0.26 | 0.23 | 0.01 | 0.04 | 7881 |
| 9 | Base + water source + sanitation + energy poverty indicators + socioeconomic variables + demographic variables + smoking in the household + (primary cooking fuel * financial security) | 0.27 | 0.23 | 0.00 | 0.04 | 7882 |
| 10 | Base + water source + sanitation + energy poverty indicators + socioeconomic variables + demographic variables + smoking in household + (land ownership * financial security) | 0.26 | 0.22 | 0.00 | 0.04 | 7895 |
| **11** | **FINAL MODEL: Base + energy poverty indicators socioeconomic variables + demographic variables + smoking in household + (education * financial security)** | **0.26** | **0.23** | **0.01** | **0.04** | **7877** |

1. Base model includes a random intercept for community

2. Energy poverty indicators included in regression model: *primary cooking fuel, electricity access for lighting (yes/no), average daily cooking time, cooking fuel decision-maker (yes/no), number of cooking-related burns experienced during the previous year, number of times collecting cooking fuel per month*

3. Demographic variables included in regression model: *age, household head (yes/no), number of household members, marital status*

4. Socioeconomic variables included in regression model: *financial security status, highest household education level, owns land (yes/no)*

a. Variance explained by random + fixed effects.

b. Variance explained by fixed effects only.

c. Intraclass correlation coefficient, representing the proportion of variability explained by between-community differences

d. Akaike information criterion

**Supplementary Table 6.** Model performance parameters for linear fixed effects model of MCS scores

| **#** | **Model** | **R^2^** | **Change in R^2^** | **AIC^a^** |
| --- | --- | --- | --- | --- |
| 1 | Base fixed effects model^1^ | 0.08 | -- | 8109 |
| 2 | Base + primary cooking fuel | 0.09 | 0.01 | 8108 |
| 3 | Base + primary cooking fuel + water source | 0.09 | 0.00 | 8102 |
| 4 | Base + energy poverty indicators^2^ | 0.22 | 0.14 | 7925 |
| 5 | Base + energy poverty indicators + water source | 0.23 | 0.01 | 7924 |
| 6 | Base + energy poverty indicators + water source + demographic variables^3^ | 0.23 | 0.00 | 7922 |
| 7 | Base + energy poverty indicators + water source + demographic variables + socioeconomic variables^4^ | 0.24 | 0.01 | 7914 |
| 8 | Base + energy poverty indicators + water source + socioeconomic variables + demographic variables + smoking in the household | 0.25 | 0.01 | 7911 |
| 9 | Base + energy poverty indicators + water source + socioeconomic variables + demographic variables + smoking in the household + (primary cooking fuel * country) | 0.25 | 0.00 | 7905 |
| 10 | Base + energy poverty indicators + water source + socioeconomic variables + demographic variables + smoking in household + (education * financial security) | 0.25 | 0.00 | 7908 |
| 11 | **FINAL MODEL: Base + energy poverty indicators + water source + socioeconomic variables + demographic variables + smoking in household + (country * financial security)** | **0.27** | **0.02** | **7873** |
| 12 | Base + energy poverty indicators + water source + socioeconomic variables + demographic variables + smoking in household + (land ownership * country * financial security) | 0.28 | 0.01 | 7874 |
| 13 | Base + energy poverty indicators + water source + socioeconomic variables + demographic variables + smoking in household + (land ownership * country * financial security) + (education * financial security) | 0.28 | -0.03 | 7876 |

1. Base model includes a fixed effect for community

2. Energy poverty indicators included in regression model: *primary cooking fuel, electricity access for lighting (yes/no), average weekly cooking time, cooking fuel decision-maker (yes/no), number of cooking-related burns experienced during the previous year, number of times collecting cooking fuel per month*

3. Demographic variables included in regression model: *age, household head (yes/no), number of household members, marital status*

4. Socioeconomic variables included in regression model: *financial security status, highest household education level, owns land (yes/no)*

a. Akaike information criterion

***Sensitivity analysis including primary and secondary cooking fuel type as predictors***

**Supplementary Table 7.** Coefficients from linear random effects models of MCS and PCS scores including primary and secondary cooking fuel as an explanatory variable

|  | **MCS model** | | | **PCS model** | | |
| --- | --- | --- | --- | --- | --- | --- |
| **Characteristic** | **Beta** | **95% CI** | **p-value** | **Beta** | **95% CI** | **p-value** |
| Primary cooking fuel |  |  |  |  |  |  |
| LPG primary | — | — |  | — | — |  |
| LPG secondary | -0.83 | -2.2, 0.55 | 0.2 | -1.71 | -3.1, -0.31 | 0.016 |
| Charcoal primary (no LPG) | -0.60 | -2.0, 0.85 | 0.4 | -0.37 | -1.8, 1.1 | 0.6 |
| Wood primary (no LPG) | -0.54 | -0.27, 0.37 | 0.4 | -0.36 | -1.7, 1.0 | 0.6 |

***Poisson regression with robust error variance for odds of likely depression***

The final model coefficients for the fixed and random effects Poisson regression models are shown in Supplementary Table 8.

**Supplementary Table 8.** Coefficients from Poisson fixed and random effects models with robust error variance of likely depression (based on MCS score <42)

|  | **Fixed effects model** | | | **Random effects model** | | |
| --- | --- | --- | --- | --- | --- | --- |
| **Characteristic** | **log(IRR)^1^** | **95% CI^2^** | **p-value** | **log(IRR)^1^** | **95% CI^2^** | **p-value** |
| Primary cooking fuel |  |  |  |  |  |  |
| LPG | — | — |  | — | — |  |
| Charcoal | 0.35 | -0.10, 0.78 | 0.13 | 0.44 | -0.10, 1.0 | 0.11 |
| Wood | 0.05 | -0.27, 0.37 | 0.8 | 0.40 | -0.25, 1.1 | 0.2 |
| Electricity access |  |  |  |  |  |  |
| Yes | — | — |  | — | — |  |
| No | 0.36 | 0.07, 0.66 | 0.014 | 0.37 | 0.07, 0.67 | 0.016 |
| Number of fuel collections per month |  |  |  |  |  |  |
| 0 | — | — |  | **—** | — |  |
| 1 | -0.90 | -1.9, -0.14 | 0.036 | -0.84 | -1.7, 0.00 | 0.050 |
| 2-5 | -0.08 | -0.69, 0.45 | 0.8 | -0.04 | -0.61, 0.53 | 0.9 |
| 6+ | -0.42 | -1.4, 0.34 | 0.3 | -0.55 | -1.4, 0.32 | 0.2 |
| Cooking fuel decision maker |  |  |  |  |  |  |
| Yes | — | — |  |  |  |  |
| No | 0.13 | -0.17, 0.42 | 0.4 | 0.12 | -0.18, 0.41 | 0.4 |
| Number of burns |  |  |  |  |  |  |
| 0 | — | — |  | — | — |  |
| 1 | 0.23 | -0.64, 0.94 | 0.6 | 0.26 | -0.51, 1.0 | 0.5 |
| 2+ | 1.1 | 0.66, 1.4 | <0.001 | 1.0 | 0.59, 1.4 | <0.001 |
| Hours cooking per week |  |  |  |  |  |  |
| 0-5 | — | — |  | — | — |  |
| 5.1-10 | 0.22 | -0.19, 0.63 | 0.3 | 0.19 | -0.21, 0.60 | 0.3 |
| 10.1-15 | 0.27 | -0.13, 0.68 | 0.2 | 0.20 | -0.19, 0.60 | 0.3 |
| 15.1-37 | 0.22 | -0.18, 0.63 | 0.3 | 0.16 | -0.23, 0.55 | 0.4 |
| Age | 0.00 | -0.01, 0.02 | 0.7 | 0.00 | -0.01, 0.02 | 0.7 |
| Martial status |  |  |  |  |  |  |
| Married | — | — |  | — | — |  |
| Living with partner | -0.10 | -0.56, 0.33 | 0.6 | -0.06 | -0.50, 0.37 | 0.8 |
| Single | -0.21 | -0.63, 0.21 | 0.3 | -0.17 | -0.58, 0.24 | 0.4 |
| Widowed | 0.26 | -0.29, 0.79 | 0.3 | 0.31 | -0.23, 0.85 | 0.3 |
| Number of household members | 0.06 | 0.00, 0.11 | 0.030 | 0.06 | 0.00, 0.11 | 0.032 |
| Household head |  |  |  |  |  |  |
| Yes | — | — |  | — | — |  |
| No | -0.08 | -0.51, 0.36 | 0.7 | -0.03 | -0.47, 0.41 | 0.9 |
| Number of rooms | -0.09 | -0.20, 0.03 | 0.2 | -0.07 | -0.19, 0.04 | 0.2 |
| Education |  |  |  |  |  |  |
| No education | — | — |  | — | — |  |
| Primary | 0.39 | -0.39, 1.4 | 0.4 | 0.48 | -0.38, 1.3 | 0.3 |
| Secondary | 0.30 | -0.47, 1.3 | 0.5 | 0.36 | -0.50, 1.2 | 0.4 |
| University | 0.41 | -0.43, 1.4 | 0.4 | 0.49 | -0.43, 1.4 | 0.3 |
| Financial security |  |  |  |  |  |  |
| Definitely not enough | — | — |  | — | — |  |
| Not quite enough | 0.26 | -0.06, 0.58 | 0.11 | 0.27 | -0.04, 0.59 | 0.090 |
| Enough to buy everything | 0.18 | -0.27, 0.62 | 0.4 | 0.17 | -0.26, 0.61 | 0.4 |
| Land owner |  |  |  |  |  |  |
| Yes | — | — |  | N/A |  |  |
| No | -0.05 | -0.39, 0.29 | 0.8 | N/A |  |  |
| Smoking inside household |  |  |  |  |  |  |
| No | — | — |  | — | — |  |
| Yes | 0.32 | -0.13, 0.73 | 0.14 | 0.32 | -0.11, 0.75 | 0.14 |
| Toilet in household |  |  |  |  |  |  |
| No | — | — |  |  |  |  |
| Yes | -0.15 | -0.47, 0.17 | 0.4 | -0.20 | -0.53, 0.14 | 0.2 |
| Primary water source |  |  |  |  |  |  |
| From river/rainwater | N/A |  |  | — | — |  |
| Communal standpipe | N/A |  |  | 0.51 | -0.46, 1.5 | 0.3 |
| Pipe in home | N/A |  |  | 0.67 | -0.26, 1.6 | 0.2 |
| Pump (deep well) | N/A |  |  | 0.51 | -0.54, 1.6 | 0.3 |
| Pit with bucket | N/A |  |  | 0.45 | -0.47, 1.4 | 0.3 |
| Country |  |  |  |  |  |  |
| Ghana | — | — |  | N/A |  |  |
| Cameroon | 1.1 | 0.53, 1.7 | <0.001 | N/A |  |  |
| Kenya | 0.90 | 0.40, 1.4 | <0.001 | N/A |  |  |
| 1IRR = Incidence Rate Ratio, CI = Confidence Interval | | | |  |  |  |

**Supplementary Table 9.** Model performance parameters for linear random effects model of PCS scores

| **#** | **Model** | **Conditional R^2 a^** | **Marginal R^2 b^** | **Change in Marginal R^2^** | **ICC^c^** | **AIC^d^** |
| --- | --- | --- | --- | --- | --- | --- |
| 1 | Base fixed effects model^1^ | 0.17 | 0.00 | -- | 0.17 | 8176 |
| 2 | Base + primary cooking fuel | 0.16 | 0.02 | 0.02 | 0.14 | 8149 |
| 3 | Base + energy poverty indicators^2^ | 0.17 | 0.04 | 0.02 | 0.15 | 8139 |
| 4 | Base + primary cooking fuel + water source | 0.23 | 0.13 | 0.09 | 0.12 | 8140 |
| 5 | Base + energy poverty indicators + water source | 0.18 | 0.04 | -0.09 | 0.15 | 8137 |
| 6 | Base + energy poverty indicators + water source + injured while collecting cooking fuel | 0.19 | 0.04 | 0.00 | 0.15 | 8130 |
| 7 | Base + energy poverty indicators + water source + injured while collecting cooking fuel + age | 0.31 | 0.16 | 0.12 | 0.18 | 7947 |
| 8 | Base + energy poverty indicators + water source + injured while collecting cooking fuel + demographic variables^3^ | 0.33 | 0.17 | 0.01 | 0.18 | 7940 |
| 9 | Base + energy poverty indicators + water source + demographic variables + socioeconomic variables^4^ | 0.31 | 0.18 | 0.01 | 0.17 | 7941 |
| 10 | Base + energy poverty indicators + water source + injured while collecting cooking fuel + socioeconomic variables + demographic variables + smoking in the household + alcohol consumption | 0.30 | 0.19 | 0.01 | 0.17 | 7940 |
| 11 | **FINAL MODEL: Base + energy poverty indicators + water source + injured while collecting cooking fuel + socioeconomic variables + demographic variables + smoking in the household + alcohol consumption + physical health condition** | **0.32** | **0.21** | **0.02** | **0.13** | **7911** |
| 12 | Base + energy poverty indicators + water source + injured while collecting cooking fuel + socioeconomic variables + demographic variables + smoking in the household + alcohol consumption + physical health condition + (education * financial security) | 0.32 | 0.21 | 0.00 | 0.13 | 7904 |
| 13 | Base + energy poverty indicators + water source + injured while collecting cooking fuel + socioeconomic variables + demographic variables + smoking in the household + alcohol consumption + physical health condition + (primary cooking fuel * financial security) | 0.32 | 0.21 | 0.00 | 0.14 | 7899 |

1. Base model includes a random intercept for community

2. Energy poverty indicators included in regression model: *primary cooking fuel, electricity access for lighting (yes/no), average daily cooking time, cooking fuel decision-maker (yes/no), number of cooking-related burns experienced during the previous year, number of times collecting cooking fuel per month*

3. Demographic variables included in regression model: *age, household head (yes/no), number of household members, marital status*

4. Socioeconomic variables included in regression model: *financial security status, highest household education level, owns land (yes/no)*

a. Variance explained by random + fixed effects.

b. Variance explained by fixed effects only.

c. Intraclass correlation coefficient, representing the proportion of variability explained by between-community differences

d. Akaike information criterion

**Supplementary Table 10.** Model performance parameters for linear fixed effects model of PCS scores

| **#** | **Model** | **R^2^** | **Change in R^2^** | **AIC^a^** |
| --- | --- | --- | --- | --- |
| 1 | Base fixed effects model^1^ | 0.12 | -- | 8166 |
| 2 | Base + primary cooking fuel | 0.14 | 0.02 | 8141 |
| 3 | Base + primary cooking fuel + water source | 0.14 | 0.00 | 8146 |
| 4 | Base + energy poverty indicators^2^ | 0.14 | 0.00 | 8152 |
| 5 | Base + energy poverty indicators + water source | 0.14 | 0.00 | 8152 |
| 6 | Base + energy poverty indicators + water source + injured while collecting cooking fuel | 0.15 | 0.01 | 8148 |
| 7 | Base + energy poverty indicators + water source + injured while collecting cooking fuel + age | 0.28 | 0.13 | 7957 |
| 8 | Base + energy poverty indicators + water source + injured while collecting cooking fuel + demographic variables^3^ | 0.29 | 0.01 | 7948 |
| 9 | Base + energy poverty indicators + water source + demographic variables + socioeconomic variables^4^ | 0.29 | 0.00 | 7955 |
| 10 | Base + energy poverty indicators + water source + injured while collecting cooking fuel + socioeconomic variables + demographic variables + smoking in the household + alcohol consumption | 0.29 | 0.00 | 7957 |
| 11 | **FINAL MODEL: Base + energy poverty indicators + water source + injured while collecting cooking fuel + socioeconomic variables + demographic variables + smoking in the household + alcohol consumption + physical health condition** | 0.31 | 0.02 | 7928 |
| 12 | Base + energy poverty indicators + water source + injured while collecting cooking fuel + socioeconomic variables + demographic variables + smoking in the household + alcohol consumption + physical health condition + (primary cooking fuel * country) | 0.31 | 0.00 | 7931 |
| 13 | Base + energy poverty indicators + water source + injured while collecting cooking fuel + socioeconomic variables + demographic variables + smoking in the household + alcohol consumption + physical health condition + (education * financial security) | 0.30 | -0.01 | 7939 |
| 14 | Base + energy poverty indicators + water source + socioeconomic variables + demographic variables + smoking in household + (injured while collecting cooking fuel * average weekly cooking time) | 0.31 | 0.01 | 7929 |
| 15 | Base + energy poverty indicators + water source + injured while collecting cooking fuel + socioeconomic variables + demographic variables + smoking in the household + alcohol consumption + physical health condition + (education * financial security) | 0.25 | -0.06 | 7939 |

1. Base model includes a fixed effect for community

2. Energy poverty indicators included in regression model: *primary cooking fuel, electricity access for lighting (yes/no), average weekly cooking time, cooking fuel decision-maker (yes/no), number of cooking-related burns experienced during the previous year, number of times collecting cooking fuel per month*

3. Demographic variables included in regression model: *age, household head (yes/no), number of household members, marital status*

4. Socioeconomic variables included in regression model: *financial security status, highest household education level, owns land (yes/no)*

a. Akaike information criterion

**Supplementary Table 11.** Coefficients from linear regression of physical component summary (PCS) score (N=1,150)

|  | **Fixed effects model** | | | **Random effects model** | |
| --- | --- | --- | --- | --- | --- |
| **Characteristic** | **Beta** | **95% CI^1^** | **p-value** | **Beta** | **95% CI^1^** |
| Primary cooking fuel |  |  |  |  |  |
| LPG | — | — |  | — | — |
| Charcoal | -0.51 | -1.8, 0.79 | 0.4 | -0.46 | -1.8, 0.83 |
| Wood | -1.1 | -2.3, 0.19 | 0.10 | -1.1 | -2.4, 0.15 |
| Electricity access |  |  |  |  |  |
| Yes | — | — |  | — | — |
| No | 0.08 | -1.0, 1.2 | 0.9 | 0.06 | -1.0, 1.2 |
| Number of fuel collections per month |  |  |  |  |  |
| 0 | — | — |  |  |  |
| 1 | -0.42 | -2.5, 1.6 | 0.7 | -0.40 | -2.4, 1.7 |
| 2-5 | 0.36 | -1.7, 2.5 | 0.7 | 0.36 | -1.7, 2.5 |
| 6+ | -0.04 | -2.4, 2.4 | >0.9 | 0.03 | -2.4, 2.4 |
| Cooking fuel decision maker |  |  |  |  |  |
| Yes | — | — |  | — | — |
| No | -0.65 | -1.8, 0.51 | 0.3 | -0.68 | -1.8, 0.47 |
| Number of burns |  |  |  |  |  |
| 0 | — | — |  | — | — |
| 1 | -1.1 | -3.9, 1.8 | 0.5 | -1.1 | -4.0, 1.8 |
| 2+ | -1.2 | -3.1, 0.75 | 0.2 | -1.2 | -3.2, 0.70 |
| Hours cooking per week |  |  |  |  |  |
| 0-5 | — | — |  | — | — |
| 5.1-10 | 1.3 | 0.01, 2.7 | 0.048 | 1.3 | 0.00, 2.7 |
| 10.1-15 | 1.5 | 0.10, 2.8 | 0.035 | 1.4 | 0.09, 2.8 |
| 15.1-37 | 1.5 | 0.11, 3.0 | 0.035 | 1.5 | 0.10, 3.0 |
| Injured while collecting cooking fuel |  |  |  |  |  |
| No | — | — |  | — | — |
| Yes | -4.8 | -8.2, -1.4 | 0.005 | -4.8 | -8.1, -1.4 |
| Age | -0.25 | -0.30, -0.20 | <0.001 | -0.25 | -0.30, -0.20 |
| Martial status |  |  |  |  |  |
| Married | — | — |  | — | — |
| Living with partner | -1.4 | -2.9, 0.21 | 0.089 | -1.4 | -3.0, 0.14 |
| Single | -0.46 | -1.8, 0.92 | 0.5 | -0.48 | -1.9, 0.90 |
| Widowed | -3.6 | -5.6, -1.6 | <0.001 | -3.6 | -5.6, -1.6 |
| Number of household members | 0.13 | -0.07, 0.32 | 0.2 | 0.12 | -0.07, 0.32 |
| Household head |  |  |  |  |  |
| Yes | — | — |  | — | — |
| No | -0.76 | -2.2, 0.63 | 0.3 | -0.77 | -2.2, 0.61 |
| Number of rooms | 0.15 | -0.15, 0.45 | 0.3 | 0.14 | -0.16, 0.44 |
| Education |  |  |  |  |  |
| No education | — | — |  | — | — |
| Primary | -1.7 | -3.9, 0.52 | 0.14 | -1.7 | -3.9, 0.51 |
| Secondary | -1.0 | -3.1, 1.1 | 0.4 | -1.0 | -3.2, 1.1 |
| University | -1.1 | -3.5, 1.3 | 0.4 | -1.1 | -3.5, 1.3 |
| Financial security |  |  |  |  |  |
| Definitely not enough | — | — |  | — | — |
| Not quite enough | -0.74 | -1.9, 0.38 | 0.2 | -0.74 | -1.9, 0.39 |
| Enough to buy everything | -0.13 | -1.5, 1.2 | 0.9 | -0.10 | -1.5, 1.3 |
| Landowner |  |  |  |  |  |
| Yes | — | — |  | — | — |
| No | -0.42 | -1.5, 0.65 | 0.4 | -0.47 | -1.5, 0.60 |
| Smoking inside household |  |  |  |  |  |
| No | — | — |  | — | — |
| Yes | -0.29 | -2.1, 1.6 | 0.8 | -0.30 | -2.1, 1.5 |
| Alcohol consumption |  |  |  |  |  |
| No | — | — |  | — | — |
| Yes | -0.94 | -2.3, 0.39 | 0.2 | -1.0 | -2.4, 0.27 |
| Presence of existing health conditions |  |  |  |  |  |
| No | — | — |  | — | — |
| Yes | -3.7 | -5.0, -2.4 | <0.001 | -3.7 | -5.0, -2.4 |
| Toilet in household |  |  |  |  |  |
| No | — | — |  | — | — |
| Yes | 0.07 | -1.0, 1.2 | 0.9 | 0.07 | -1.0, 1.2 |
| Water source |  |  |  |  |  |
| Collect from river | — | — |  | — | — |
| Communal standpipe | -2.6 | -5.4, 0.24 | 0.073 | -2.6 | -5.4, 0.24 |
| Pipe in home | -2.0 | -4.7, 0.70 | 0.15 | -2.0 | -4.7, 0.68 |
| Pump (deep well) | -0.78 | -3.9, 2.4 | 0.6 | -0.79 | -3.9, 2.4 |
| Well (pit with bucket) | -3.1 | -5.7, -0.47 | 0.021 | -3.1 | -5.7, -0.48 |
| Country |  |  |  |  |  |
| Ghana | — | — |  | N/A |  |
| Cameroon | -5.4 | -7.3, -3.6 | <0.001 | N/A |  |
| Kenya | -0.45 | -2.0, 1.1 | 0.6 | N/A |  |

***Burn severity***

**Supplementary Table 12**. Number of children under 5 years by number of cooking-related burns in the previous year

| **Number of cooking-related burns** | **Number of children under 5** | |
| --- | --- | --- |
|  | **0** | **1 or more** |
| **0** | 337 (89%) | 703 (90%) |
| **1** | 6 (1%) | 22 (3%) |
| **2+** | 36 (10%) | 52 (7%) |

**Supplementary Table 13.** Scarring reported by those experiencing cooking-related burns

| **Level of scarring** | **N (%)** |
| --- | --- |
| No scar | 33 (28%) |
| Small scar (smaller than nickel) | 75 (64%) |
| Large scar (size of nickel or larger) | 9 (8%) |

***Evaluating water source as a confounder between energy poverty and health-related quality of life***

**Supplementary Table 14.** Beta coefficients from Poisson regression with robust error variance assessing odds of depression. Only primary cooking fuel variable included.

| Characteristic | log(IRR)1 | 95% CI1 | p-value |
| --- | --- | --- | --- |
| **Primary cooking fuel** |  |  |  |
| Charcoal | — | — |  |
| LPG | -0.42 | -0.84, 0.01 | 0.05 |
| Wood | -0.40 | -0.85, 0.05 | 0.08 |
| **Community** |  |  |  |
| Obuasi, Ghana | — | — |  |
| Mbalmayo, Cameroon | 1.6 | 1.2, 2.1 | <0.001 |
| Eldoret, Kenya | 0.91 | 0.49, 1.4 | <0.001 |
| 1IRR = Incidence Rate Ratio, CI = Confidence Interval | | | |

**Supplementary Table 15.** Beta coefficients from Poisson regression with robust error variance assessing odds of depression. Primary cooking fuel and main household water source variables included.

| Characteristic | log(IRR)1 | 95% CI1 | p-value |
| --- | --- | --- | --- |
| **Primary cooking fuel** |  |  |  |
| Charcoal | — | — |  |
| LPG | -0.43 | -0.85, 0.00 | 0.04* |
| Wood | -0.28 | -0.73, 0.18 | 0.20 |
| **Primary water source** |  |  |  |
| Collect from river | — | — |  |
| Communal standpipe | 0.52 | -0.35, 1.6 | 0.30 |
| Pipe in home | 0.78 | -0.02, 1.8 | 0.09 |
| Pump (deep well) | 0.39 | -0.58, 1.5 | 0.50 |
| Well (pit with bucket) | 0.32 | -0.49, 1.4 | 0.50 |
| **Community** |  |  |  |
| Obuasi, Ghana | — | — |  |
| Mbalmayo, Cameroon | 1.6 | 1.1, 2.0 | <0.001 |
| Eldoret, Kenya | 0.94 | 0.49, 1.4 | <0.001 |
| 1IRR = Incidence Rate Ratio, CI = Confidence Interval | | | |

***Sensitivity analysis including body mass index as a predictor***

**Supplementary Table 16.** Coefficients from linear random effects regression of mental component summary (MCS) score including body mass index (BMI) (N=958)

|  | **Model with BMI** | | **Original model without BMI** | |
| --- | --- | --- | --- | --- |
| **Characteristic** | **Beta** | **95% CI** | **Beta** | **95% CI** |
| Primary cooking fuel |  |  |  |  |
| LPG | — | — | — | — |
| Charcoal | -0.64 | -1.9, 0.66 | -0.89 | -2.2, 0.40 |
| Wood | -0.42 | -1.8, 0.94 | -0.43 | -1.7, 0.82 |
| **BMI category** |  |  |  |  |
| **Underweight** | **-1.4** | **-4.2, 1.5** | N/A |  |
| **Normal weight** | **—** | **—** | N/A |  |
| **Obese** | **0.91** | **-0.32, 2.1** | N/A |  |
| **Overweight** | **0.37** | **-0.79, 1.5** | N/A |  |
| Electricity access |  |  |  |  |
| Yes | — | — | — | — |
| No | -2.3 | -3.4, -1.1 | -2.6 | -3.7, -1.5 |
| Number of fuel collections per month |  |  |  |  |
| 0 | **—** | — | **—** | — |
| 1 | 2.8 | 0.61, 5.0 | 2.2 | 0.17, 4.2 |
| 2-5 | 2.0 | -0.15, 4.1 | 0.93 | -1.1, 2.9 |
| 6+ | -0.70 | -3.1, 1.8 | -1.0 | -3.3, 1.3 |
| Cooking fuel decision maker |  |  |  |  |
| Yes | — | — | **—** | — |
| No | -0.54 | -1.8, 0.70 | -0.79 | -1.9, 0.36 |
| Number of burns |  |  |  |  |
| 0 | — | — | — | — |
| 1 | 0.06 | -3.0, 3.1 | -1.0 | -3.8, 1.8 |
| 2+ | -9.6 | -12, -7.5 | -9.4 | -11, -7.5 |
| Hours cooking per week |  |  |  |  |
| 0-5 | — | — | — | — |
| 5.1-10 | -1.3 | -2.7, 0.12 | -0.85 | -2.2, 0.47 |
| 10.1-15 | -2.8 | -4.3, -1.3 | -1.9 | -3.3, -0.61 |
| 15.1-37 | -3.0 | -4.6, -1.5 | -2.4 | -3.8, -1.0 |
| Age | 0.00 | -0.05, 0.05 | 0.00 | -0.05, 0.05 |
| Martial status |  |  |  |  |
| Married | — | — | — | — |
| Living with partner | -0.39 | -2.0, 1.3 | -0.06 | -1.6, 1.5 |
| Single | 1.1 | -0.33, 2.6 | 0.64 | -0.72, 2.0 |
| Widowed | -0.61 | -2.7, 1.5 | -0.92 | -2.9, 1.1 |
| Number of household members | -0.17 | -0.38, 0.04 | -0.17 | -0.36, 0.02 |
| Household head |  |  |  |  |
| Yes | — | — | — | — |
| No | -2.0 | -3.4, -0.49 | 1.3 | -0.05, 2.7 |
| Number of rooms | 0.06 | -0.25, 0.37 | 0.12 | -0.18, 0.42 |
| Education |  |  |  |  |
| No education | — | — | — | — |
| Primary | -0.03 | -4.4, 4.4 | -0.43 | -4.5, 3.7 |
| Secondary | 0.54 | -3.9, 4.9 | 1.1 | -3.0, 5.2 |
| University | 2.3 | -2.7, 7.4 | 3.3 | -1.5, 8.0 |
| Financial security |  |  |  |  |
| Definitely not enough | — | — | — | — |
| Not quite enough | 4.2 | -0.78, 9.2 | 4.6 | -0.23, 9.4 |
| Enough to buy everything | 4.5 | -1.0, 10.0 | 5.0 | -0.23, 10 |
| Landowner |  |  |  |  |
| Yes | — | — | — | — |
| No | -0.07 | -1.2, 1.0 | 0.08 | -1.0, 1.1 |
| Smoking inside household |  |  |  |  |
| No | — | — | — | — |
| Yes | -3.6 | -5.7, -1.6 | -2.3 | -4.1, -0.45 |
| Toilet in household |  |  |  |  |
| No | — | — |  |  |
| Yes | -0.74 | -1.9, 0.43 | 0.71 | -0.38, 1.8 |
| Water source |  |  |  |  |
| Collect from river | — | — | — | — |
| Communal standpipe | -2.6 | -5.6, 0.29 | -2.4 | -5.2, 0.40 |
| Pipe in home | -2.3 | -5.1, 0.51 | -2.1 | -4.8, 0.54 |
| Pump (deep well) | -2.4 | -5.7, 0.91 | -2.5 | -5.7, 0.60 |
| Well (pit with bucket) | -1.4 | -4.1, 1.3 | -1.0 | -3.6, 1.6 |
| Education * Financial security |  |  |  |  |
| Primary * Not quite enough | -3.9 | -9.3, 1.4 | -3.6 | -8.8, 1.5 |
| Secondary * Not quite enough | -4.7 | -9.9, 0.57 | -5.0 | -10, -0.03 |
| University * Not quite enough | -5.8 | -12, 0.04 | -6.6 | -12, -1.0 |
| Primary * Enough to buy everything | -3.6 | -9.6, 2.5 | -3.1 | -8.9, 2.7 |
| Secondary * Enough to buy everything | -1.5 | -7.3, 4.3 | -2.5 | -8.0, 3.0 |
| University * Enough to buy everything | -6.5 | -13, -0.03 | -7.6 | -14, -1.4 |

**Supplementary Table 17.** Coefficients from linear regression of physical component summary (PCS) score including body mass index (BMI) (N=958)

|  | **Model with BMI** | | **Original model without BMI** | |
| --- | --- | --- | --- | --- |
| **Characteristic** | **Beta** | **95% CI^1^** | **Beta** | **95% CI^1^** |
| Primary cooking fuel |  |  |  |  |
| LPG | — | — | — | — |
| Charcoal | -0.24 | -1.5, 1.0 | -0.46 | -1.8, 0.83 |
| Wood | -0.60 | -1.9, 0.74 | -1.1 | -2.4, 0.15 |
| BMI category |  |  |  |  |
| Normal weight | — | — |  |  |
| Underweight | -1.7 | -4.6, 1.1 |  |  |
| Obese | -1.1 | -2.3, 0.14 |  |  |
| Overweight | -1.0 | -2.1, 0.18 |  |  |
| Electricity access |  |  |  |  |
| Yes | — | — | — | — |
| No | 0.38 | -0.76, 1.5 | 0.06 | -1.0, 1.2 |
| Number of fuel collections per month |  |  |  |  |
| 0 | — | — |  |  |
| 1 | -0.89 | -3.1, 1.3 | -0.40 | -2.4, 1.7 |
| 2-5 | 0.56 | -1.6, 2.7 | 0.36 | -1.7, 2.5 |
| 6+ | -0.35 | -2.8, 2.1 | 0.03 | -2.4, 2.4 |
| Cooking fuel decision maker |  |  |  |  |
| Yes | — | — | — | — |
| No | -0.91 | -2.1, 0.30 | -0.68 | -1.8, 0.47 |
| Number of burns |  |  |  |  |
| 0 | — | — | — | — |
| 1 | -1.8 | -4.8, 1.2 | -1.1 | -4.0, 1.8 |
| 2+ | -1.0 | -3.0, 1.0 | -1.2 | -3.2, 0.70 |
| Hours cooking per week |  |  |  |  |
| 0-5 | — | — | — | — |
| 5.1-10 | 1.0 | -0.44, 2.4 | 1.3 | 0.00, 2.7 |
| 10.1-15 | 0.35 | -1.1, 1.8 | 1.4 | 0.09, 2.8 |
| 15.1-37 | 0.42 | -1.1, 2.0 | 1.5 | 0.10, 3.0 |
| Injured while collecting cooking fuel |  |  |  |  |
| No | — | — | — | — |
| Yes | -4.8 | -8.2, -1.4 | -4.8 | -8.1, -1.4 |
| Age | -0.22 | -0.27, -0.16 | -0.25 | -0.30, -0.20 |
| Martial status |  |  |  |  |
| Married | — | — | — | — |
| Living with partner | -1.8 | -3.4, -0.17 | -1.4 | -3.0, 0.14 |
| Single | -0.72 | -2.1, 0.69 | -0.48 | -1.9, 0.90 |
| Widowed | -3.6 | -5.6, -1.5 | -3.6 | -5.6, -1.6 |
| Number of household members | 0.02 | -0.19, 0.22 | 0.12 | -0.07, 0.32 |
| Household head |  |  |  |  |
| Yes | — | — | — | — |
| No | 0.59 | -0.84, 2.0 | -0.77 | -2.2, 0.61 |
| Number of rooms | 0.25 | -0.06, 0.55 | 0.14 | -0.16, 0.44 |
| Education |  |  |  |  |
| No education | — | — | — | — |
| Primary | -1.8 | -4.0, 0.42 | -1.7 | -3.9, 0.51 |
| Secondary | -1.4 | -3.5, 0.74 | -1.0 | -3.2, 1.1 |
| University | -1.9 | -4.3, 0.57 | -1.1 | -3.5, 1.3 |
| Financial security |  |  |  |  |
| Definitely not enough | — | — | — | — |
| Not quite enough | -1.3 | -2.4, -0.08 | -0.74 | -1.9, 0.39 |
| Enough to buy everything | -0.42 | -1.8, 1.0 | -0.10 | -1.5, 1.3 |
| Landowner |  |  |  |  |
| Yes | — | — | — | — |
| No | 0.04 | -1.0, 1.1 | -0.47 | -1.5, 0.60 |
| Smoking inside household |  |  |  |  |
| No | — | — | — | — |
| Yes | 0.05 | -2.0, 2.1 | -0.30 | -2.1, 1.5 |
| Alcohol consumption |  |  |  |  |
| No | — | — | — | — |
| Yes | -0.35 | -1.8, 1.1 | -1.0 | -2.4, 0.27 |
| Presence of existing health conditions |  |  |  |  |
| No | — | — | — | — |
| Yes | -4.2 | -5.5, -2.8 | -3.7 | -5.0, -2.4 |
| Toilet in household |  |  |  |  |
| No | — | — | — | — |
| Yes | -0.63 | -1.8, 0.52 | 0.07 | -1.0, 1.2 |
| Water source |  |  |  |  |
| Collect from river | — | — | — | — |
| Communal standpipe | -2.6 | -5.5, 0.27 | -2.6 | -5.4, 0.24 |
| Pipe in home | -1.9 | -4.7, 0.83 | -2.0 | -4.7, 0.68 |
| Pump (deep well) | -0.68 | -3.9, 2.6 | -0.79 | -3.9, 2.4 |
| Well (pit with bucket) | -3.0 | -5.7, -0.38 | -3.1 | -5.7, -0.48 |

***Assessing representativeness of Phase 2 surveys in CLEAN-Air(Africa)***

**Supplementary Table 18**. Comparison of socioeconomic and cooking environment characteristics of participants receiving Phase 2 survey (n=1,223) and entire CLEAN-Air(Africa) study sample receiving Phase 1 survey (n=4,555)

| **Characteristic** | **CLEAN-Air(Africa) Phase 2 study sample**  **(N=1,223)** | **CLEAN-Air(Africa) Phase 1 study sample**  **(N=4,555)** |
| --- | --- | --- |
| Age of household head (Mean (SD)) | 35 (12) | 37 (13) |
| Fuel decision maker of household |  |  |
| Yes | 923 (80%) | 3449 (76%) |
| Marital status |  |  |
| Married | 667 (54%) | 2243 (49%) |
| Single | 289 (24%) | 1267 (28%) |
| Cohabitating | 153 (13%) | 598 (13%) |
| Widowed/divorced | 98 (8%) | 447 (10%) |
| Home size (# of members) |  |  |
| 1-2 | 141 (12%) | 652 (14%) |
| 3-4 | 382 (32%) | 1328 (29%) |
| 5-6 | 392 (32%) | 1340 (29%) |
| 7+ | 292 (24%) | 1234 (27%) |
| Primary cooking fuel type |  |  |
| LPG | 576 (47%) | 1260 (28%) |
| Wood | 413 (34%) | 1592 (35%) |
| Charcoal | 233 (19%) | 1526 (34%) |
| Electricity connection |  |  |
| Yes | 875 (72%) | 3264 (72%) |
| Household income quartile |  |  |
| 1 (lowest) | 232 (19%) | 739 (16%) |
| 2 | 259 (21%) | 770 (17%) |
| 3 | 226 (18%) | 826 (18%) |
| 4 (highest) | 216 (18%) | 557 (12%) |
| Don’t know/won’t answer | 290 (24%) | 875 (19%) |
| Obtain cooking fuels for free |  |  |
| Yes | 196 (16%) | 1189 (26%) |
| Highest HH education level |  |  |
| No formal education | 70 (6%) | 289 (6%) |
| Primary | 182 (15%) | 899 (20%) |
| Secondary/high school | 618 (50%) | 2521 (55%) |
| University | 213 (17%) | 227 (11%) |
| Toilet or septic tank in home |  |  |
| Yes | 409 (33%) | 1148 (25%) |

**Supplementary Table 19.** Physical health characteristics of female participants by community

| **Characteristic** | **Overall**  **(N=1,157)** | **Mbalmayo,**  **Cameroon**  **(N=404)** | **Obuasi, Ghana**  **(N=348)** | **Eldoret, Kenya**  **(N=405)** |
| --- | --- | --- | --- | --- |
| Smoking in the household | 70 (6%) | 38 (12%) | 15 (4%) | 17 (4%) |
| Yes |  |  |  |  |
| Consume alcohol |  |  |  |  |
| Yes | 301 (26%) | 263 (65%) | 18 (4%) | 20 (5%) |
| Presence of physical condition |  |  |  |  |
| Yes | 166 (14%) | 66 (16%) | 44 (13%) | 14 (14%) |
| Body mass index (BMI) (N=1,028) |  |  |  |  |
| Underweight | 28 (3%) | 11 (4%) | 6 (2%) | 11 (3%) |
| Normal | 318 (31%) | 101 (36%) | 91 (26%) | 125 (31%) |
| Overweight | 353 (34%) | 91 (32%) | 132 (38%) | 128 (32%) |
| Obese | 329 (32%) | 77 (27%) | 115 (33%) | 136 (34%) |

***Model cross validation***

**Supplementary Table 20.** Goodness of fit statistics for linear fixed effect models

| **Model** | **Model** | **Main model performance** | | **Cross Validation** | |
| --- | --- | --- | --- | --- | --- |
|  |  | **R^2^** | **RMSE** | **R^2^** | **RMSE** |
| Mental (MCS) | *MCS score = Base + energy poverty indicators + water source + socioeconomic variables + demographic variables + smoking in household + (country * financial security)* | 0.27 | 7.07 | 0.25 | 7.57 |
| Physical (PCS) | *PCS score = Base + energy poverty indicators + water source + injured while collecting cooking fuel + socioeconomic variables + demographic variables + smoking in the household + alcohol consumption + physical health condition* | 0.31 | 7.11 | 0.25 | 7.82 |

RMSE= root mean squared error

Base model includes a fixed effect for community

Energy poverty indicators included in regression model: *primary cooking fuel, electricity access for lighting (yes/no), average weekly cooking time, cooking fuel decision-maker (yes/no), number of cooking-related burns experienced during the previous year, number of times collecting cooking fuel per month*

Demographic variables included in regression model: *age, household head (yes/no), number of household members, marital status*

Socioeconomic variables included in regression model: *financial security status, highest household education level, owns land (yes/no)*

***Cooking-related burns in Mbalmayo, Cameroon***

**Supplementary Figure 12.** Prevalence of cooking-related burns by primary cooking fuel type and community

***
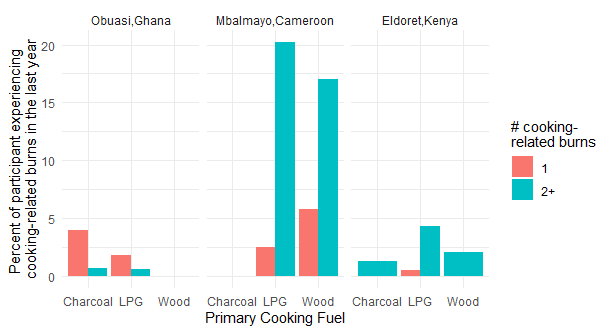
***

A monotonically decreasing relationship between increasing frequency of cooking-related burns and MCS was found among participants primarily cooking with wood but not with LPG in Mbalmayo (Supplementary Figure 15).

**Supplementary Figure 13.** Median and interquartile range of MCS scores by cooking fuel type and number of cooking-related burns in Mbalmayo, Cameroon


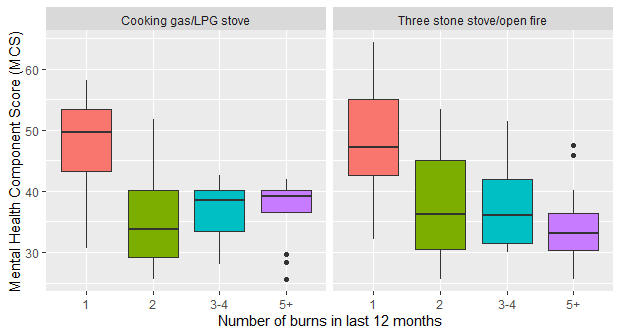


**Supplementary Table 21.** Model performance parameters for Poisson fixed effects model with robust error variance assessing odds of likely depression

| **#** | **Model** | **R^2 a^** | **Change in R^2 a^** | **AIC ^b^** |
| --- | --- | --- | --- | --- |
| 1 | Base fixed effects model^1^ | 0.12 | -- | 1208 |
| 2 | Base + primary cooking fuel | 0.12 | 0.00 | 1209 |
| 3 | Base + primary cooking fuel + water source | 0.14 | 0.02 | 1205 |
| 4 | Base + energy poverty indicators^2^ | 0.25 | 0.11 | 1153 |
| 5 | Base + energy poverty indicators + water source | 0.25 | 0.00 | 1158 |
| 6 | Base + energy poverty indicators + water source + sanitation | 0.25 | 0.00 | 1157 |
|  | Base + energy poverty indicators + sanitation | 0.25 | 0.00 | 1154 |
| **7** | Base + energy poverty indicators + sanitation + demographic variables^3^ | 0.27 | 0.02 | 1160 |
| 8 | Base + energy poverty indicators + sanitation + demographic variables + socioeconomic variables^4^ | 0.27 | 0.00 | 1162 |
| 9 | **FINAL MODEL:** **Base + energy poverty indicators + sanitation + socioeconomic variables + demographic variables + smoking in the household** | **0.28** | **0.01** | **1164** |
| 10 | Base + energy poverty indicators + sanitation + socioeconomic variables + demographic variables + (primary cooking fuel * financial security) | 0.28 | 0.00 | 1169 |

1. Base model includes a fixed effect for community

2. Energy poverty indicators included in regression model: *primary cooking fuel, electricity access for lighting (yes/no), average daily cooking time, cooking fuel decision-maker (yes/no), number of cooking-related burns experienced during the previous year, number of times collecting cooking fuel per month*

3. Demographic variables included in regression model: *age, household head (yes/no), number of household members, marital status*

4. Socioeconomic variables included in regression model: *financial security status, highest household education level,*

a. Represents the Nagelkerke’s pseudo R^2^

b. Akaike information criterion
